# Impaired cerebral microvascular endothelial cells integrity due to elevated dopamine in myasthenic model

**DOI:** 10.1186/s12974-023-03005-3

**Published:** 2024-01-04

**Authors:** Yue Hao, Yinchun Su, Yifan He, Wenyuan Zhang, Yang Liu, Yu Guo, Xingfan Chen, Chunhan Liu, Siyu Han, Buyi Wang, Yushuang Liu, Wei Zhao, Lili Mu, Jinghua Wang, Haisheng Peng, Junwei Han, Qingfei Kong

**Affiliations:** 1 Department of Neurobiology, Harbin Medical University, Heilongjiang Provincial Key Laboratory of Neurobiology, Harbin, 150081 Heilongjiang China; 2 Medicine Department of Guangzhou Geriatric Hospital, Guangzhou, 510260 Guangdong China; 3 Department of Pharmacology, School of Medicine, Shaoxing University, Shaoxing, 312000 Zhejiang China; 4 College of Bioinformatics Science and Technology, Harbin Medical University, Harbin, 150081 Heilongjiang China; 5 The Heilongjiang Provincial Joint Laboratory of Basic Medicine and Multiple Organ System Diseases (International Cooperation), Harbin, 150081 Heilongjiang China

**Keywords:** Dopamine, Endothelial cells, Central nervous system, Tight junction, Adherence junctions, Wnt/β-catenin pathway, Experimental autoimmune myasthenia gravis

## Abstract

Myasthenia gravis is an autoimmune disease characterized by pathogenic antibodies that target structures of the neuromuscular junction. However, some patients also experience autonomic dysfunction, anxiety, depression, and other neurological symptoms, suggesting the complex nature of the neurological manifestations. With the aim of explaining the symptoms related to the central nervous system, we utilized a rat model to investigate the impact of dopamine signaling in the central nervous and peripheral circulation. We adopted several screening methods, including western blot, quantitative PCR, mass spectrum technique, immunohistochemistry, immunofluorescence staining, and flow cytometry. In this study, we observed increased and activated dopamine signaling in both the central nervous system and peripheral circulation of myasthenia gravis rats. Furthermore, changes in the expression of two key molecules, Claudin5 and CD31, in endothelial cells of the blood–brain barrier were also examined in these rats. We also confirmed that dopamine incubation reduced the expression of ZO1, Claudin5, and CD31 in endothelial cells by inhibiting the Wnt/β-catenin signaling pathway. Overall, this study provides novel evidence suggesting that pathologically elevated dopamine in both the central nervous and peripheral circulation of myasthenia gravis rats impair brain–blood barrier integrity by inhibiting junction protein expression in brain microvascular endothelial cells through the Wnt/β-catenin pathway.

**Graphical Abstract:**

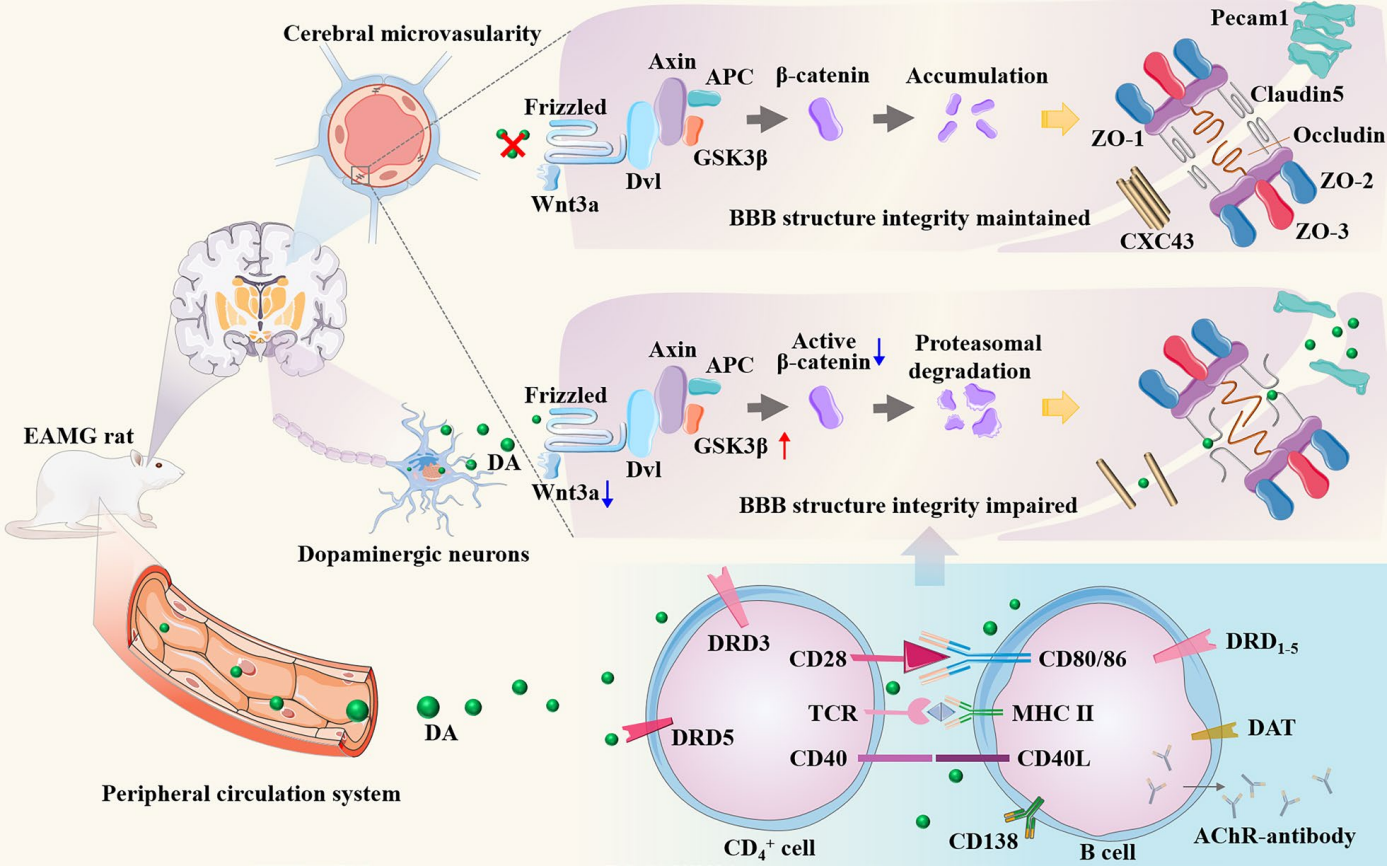

**Supplementary Information:**

The online version contains supplementary material available at 10.1186/s12974-023-03005-3.

## Background

Myasthenia gravis (MG) is an autoimmune inflammatory disorder primarily involving B cell-mediated T cell response. It is characterized by the blocking of neuromuscular transmission through the presence of antibodies, resulting in skeletal muscle weakness and rapid muscle fatigue [1–3]. While MG predominantly affects the neuromuscular system, growing evidence has also suggested the occurrence of central nervous system (CNS) disorders in a subset of patients [4, 5]. These individuals may experience various neurological symptoms, including headaches, sleep disorders, dysautonomia, depression, anxiety disorders, and cognitive and psychosocial issues [6–8]. Neuropsychological tests have shown a significant association between MG severity and depression as well as chronic stress [9, 10]. Furthermore, cognitive fatigue has been observed through repeated testing of attention and concentration [8]. Abnormal electroencephalograph recordings occurred in 14 of 118 MG patients (with eight diffuse slow abnormalities and six focal slow abnormalities), indicating disturbances were involved in the CNS [11]. Clinical reports have demonstrated an increased percentage of T lymphocytes and elevated IgG concentrations in cerebrospinal fluid (CSF) in 12 MG patients [12, 13], further supporting the change of the CNS in some MG cases. Therefore, viewing MG as a pure muscular motor manifestation hinders further research into its pathogenesis. However, the mechanisms by which the CNS is involved in MG remain incompletely understood.

The blood–brain barrier (BBB) is a vital physiological barrier that separates the CNS from the peripheral circulation. It regulates the nutrients transfer and prevents the entry of metabolic wastes into the brain [14, 15]. The BBB is a multicellular vascular structure formed by endothelial cells (ECs), pericytes (PCs), and glial cells. ECs play a primary role in strictly controlling the passage of substances into the CNS by forming four types of complex and continuous junctions: tight junctions (TJ: primarily consisting of zonula occludens-1 (ZO-1), ZO-2, ZO-3, and Claudin5) [16], adherence junctions (AJ: including VE-cadherin and platelet ECs adhesion molecule-1 (PECAM-1), also known as cluster of differentiation 31 (CD31)) [16], gap junctions (connexin-37, connexin-40, and connexin-43) [17], and junctional adhesion molecules [18]. Among these, the expression of ZO1, Claudin5, and CD31 by ECs is indispensable for maintaining BBB integrity. Suppressing these junction expressions in cerebral microvascular ECs leads to the subsequent BBB integrity damage. This allows the entry of inflammatory cytokines and harmful molecules into the brain through the compromised BBB, resulting in the CNS lesions associated with diseases such as multiple sclerosis (MS) and Alzheimer’s disease [19, 20]. The Wnt/β-catenin pathway is considered an essential signaling pathway for maintaining BBB integrity. It controls embryonic development and adult life in all animals through the transcriptional coactivator β-catenin [21]. The endothelial Unc5B receptor controls BBB integrity by maintaining Wnt/β-catenin signaling [22]. Activation of the Wnt/β-catenin signaling pathway enhances barrier characteristics and reduces the inflammatory phenotype in endothelial progenitor cells derived from MS [23]. Additionally, it mitigates BBB malfunction in the brain endothelium derived from Alzheimer’s disease [24].

Dopamine (DA) is an essential neurotransmitter synthesized and secreted by the substantia nigra and ventral tegmental area. It mediates various neurological functions in the CNS, including movement, reward, emotion, learning, memory, and cognition [25, 26] by binding to DA receptors (DRDs) encompassing DRD1 to DRD5 [27]. Dopaminergic neuron deletion in the substantia nigra causes movement disorders in Parkinson’s disease (PD), such as rigidity, resting tremor, and bradykinesia [28, 29]. Recent studies showed that the upregulation of dopamine D2 receptor (DRD2) in reactive astrocytes in the brains of MS patients [30], and the dysregulation of the dopaminergic system causes psychiatric symptoms in these patients [31]. These findings indicate that DA signaling is implicated in the CNS activation in MS. Additionally, DA can also exert other physiological and pathological functions in the CNS, including regulation of endothelial [32] and immune cells [33]. Studies have demonstrated that increased DA in the periphery inhibits angiotensin receptor type 1 expression in ECs, impairing angiogenesis in post-ischemic healing conditions [34]. Furthermore, DA strongly and selectively inhibits vascular permeability and angiogenic activities induced by vascular permeability factor/vascular endothelial growth factor [32]. Moreover, DA is an important immunomodulator. DRD3 plays a crucial role in CD4^+^ T cell differentiation and inflammatory progression promotion by activating Th1/Th17-mediated immunity [35]. Notably, DA derived from follicular T helper (Th) cells could bind to DA receptors on B cells to induced T cell–B cell interactions, enhancing the formation of productive synapses in germinal centers [36], and high expression of DRD1 in B cells has been positively correlated with disease progression and severity in women with rheumatoid arthritis [37].

Considering the CNS symptoms involved in MG such as headache, anxiety, and depression, as well as increased IgG in CSF and abnormal EEGs in some MG patients, we propose that DA may contribute to the CNS alternations of MG. This aspect has received limited attention in previous research, despite the significant role that DA plays in regulating CNS diseases such as PD and MS. Our investigation aims to explore the potential link between elevated DA levels in the CNS and peripheral circulation, and direct damage to cerebral microvascular ECs through Wnt/β-catenin signaling pathways. This disruption of the BBB microstructure could potentially account for the manifestation of neurological symptoms in certain MG patients. These findings shed light on the possibility of DA as a novel target for treatment in MG research.

## Materials and methods

### Animals

Female Lewis rats aged six to eight weeks and weighing 140 to 160 g were purchased from Vital River Laboratory Animal Co., Ltd. (Beijing, China). The study was conducted in compliance with the principles outlined in Harbin Medical University’s Guide to the Care and Use of Laboratory Animals, as published by the China National Institute of Health.

### Induction and clinical assessment of EAMG

Lewis rats were randomly assigned to the complete Freund’s adjuvant (CFA) group or the experimental autoimmune myasthenia gravis (EAMG) group. Lewis rats are used in the majority of contemporary MG models because they are identified as a recognized representation of MG [38]. They were then immunized with the same dose (200 μL/rat) of immune emulsion at the root of the tail. In the immune emulsion for each CFA rat, there was 100 μL of incomplete Freund’s adjuvant (IFA, Sigma Aldrich, St Louis, MO), 2 mg of Mycobacterium tuberculosis (TB, Difco, Detroit, MI), and 100 μL of phosphate-buffered saline (PBS). The only difference between the CFA and EAMG groups was the addition of R-AChR_97-116_ peptide (50 μg/rat; synthesized by AC Scientific, Inc. Xi’an, China) in the immune emulsion, which was dissolved in PBS prior to preparing the emulsion for the EAMG group. On day 30 after the first immunization, all rats received a second immunization with the same emulsion minus the TB component. Throughout the study, the body weight and clinical scores were monitored every other day. Clinical scores were measured on a scale ranging from 0 (asymptomatic) to 4 (death status) using the standardized criteria provided in Additional file 1: Table S1 [38]. We averaged the data for each animal at each time point and recorded them.

### Cell line and cell culture

The bEnd.3 cells, an immortalized mouse brain EC line, were cultured in DMEM containing 10% FBS (AusGeneX) and cultured at 37 °C with 5% CO_2_. Subsequently, the CD4^+^ T cells and B220^+^ B cells were isolated by performing a negative selection with a MagCellect Rat CD4^+^ T-Cell Isolation Kit and a MagCellect Rat B-Cell Isolation Kit (R&D Systems, Inc., USA) according to the manufacturer’s instructions. A total of 1 × 10^8^ EAMG rat splenocytes were prepared for isolation, resulting in the collection of 2–3.5 × 10^7^ CD4^+^ T lymphocytes and B cells after purification. These purified cells and lymphocytes were then recall stimulated with 10 μg/mL R-AChR_97–116_ in RPMI 1640 medium (Sigma-Aldrich) supplemented with 1% sodium pyruvate (Sigma, USA), 10% fetal bovine serum, 1% nonessential amino acids (Sigma, USA), 1% L-glutamine (Sigma-Aldrich), 1% penicillin–streptomycin (Gibco, Paisley, UK), and 2-mercaptoethanol (2-ME, Amresco, Solon, OH, USA) for an additional 48 h of in vitro incubation.

### Proteomics analysis

Protein samples for analysis were prepared from total brain tissues from CFA and EAMG rats after secondary immunization using liquid nitrogen cryopreserved. These samples were transported by dry-ice to Jingjie PTM BioLab (Hangzhou) Co., Ltd for mass spectrum analysis. Additionally, brain tissues were also prepared for western blot analysis.

After the samples were shipped to Jingjie BioLab, the samples were dissolved and digested into peptides. The peptides were analyzed in Orbitrap Exploris 480 (ThermoFisher Scientific) with a nano-electrospray ion source. The electrospray voltage applied was 2300 V. FAIMS compensate voltage (CV) was set as −45 V. Precursors and fragments were analyzed at the Orbitrap detector. The full MS scan resolution was set to 60,000 for a scan range of 400–1200 m/z. The MS/MS scan was fixed first mass as 110 m/z at a resolution of 15,000 with the TurboTMT was set as ON. Up to 25 most abundant precursors were then selected for further MS/MS analyses with 30 s dynamic exclusion. The HCD fragmentation was performed at a normalized collision energy (NCE) of 35%. Automatic gain control (AGC) target was set at 100%, with an intensity threshold of 10,000 ions/s and a maximum injection time of Auto. The resulting MS/MS data were processed using Proteome Discoverer (v2.4.1.15).

Before proceeding with further analysis, we performed a precheck using t-SNE, a dimensional reduction method to assess the separability of samples into normal and disease groups. The method of t-SNE is a widely used technique for visualizing high-dimensional data in a lower-dimensional space and has been extensively employed in exploratory analyses in many fields. To this end, we adopted Rtsne package to visualize the t-SNE result [39, 40]. By applying t-SNE to our data, we generated a two-dimensional plot that depicted the relationships between the samples in a reduced space.

Subsequently, we annotated the proteins according to their expression to identify genes associated with CNS. We utilized the Kyoto Encyclopedia of Genes and Genomes (KEGG), a widely used comprehensive resource containing detailed pathways and metabolic information. Additionally, we also incorporated Gene Ontology (GO), a standardized vocabulary for consistently and systematically describing gene and protein functions. Furthermore, we performed gene set enrichment analysis (GSEA) to identify overrepresented pathways to gain insights into the mechanisms under investigation. The enriched pathways were ranked based on the absolute value of the normalized enrichment score (NES). To compare two groups (CFA vs EAMG), we employed Student’s *t* tests. The heatmaps display row-normalized expression values.

### Isolation and identification of the cerebral microvasculature

The cerebral microvasculature was isolated following previously described methods [41]. Briefly, whole brains were collected and rinsed in ice-cold phosphate-buffered saline (PBS), from which the leptomeninges, olfactory bulb, cerebellum, and white matter were removed while preserving the cortex on ice. The remaining cortices were then homogenized in 1–2 mL of PBS using a 5 mL Dounce tissue grinder at a steady pace followed by a 2000 *g* centrifugation for 5 min at 4 °C. The resulting pellet was resuspended in 15% (wt/vol) dextran-PBS before another centrifugation at 10,000 *g* for 15 min at 4 °C. Subsequently, the pellet containing red microvasculature was suspended in 10–20 mL of PBS containing 0.5% (wt/vol) bovine serum albumin and centrifuged at 5000 *g* for 10 min at 4 °C. The resulting purified cerebral microvasculature was confirmed via HE staining for tubular structures and by assessing the absence of neuronal markers (*Syp* and *Tubb3* genes) using qPCR. This pellet was further processed for identification, as well as protein and RNA extraction.

### Immunofluorescence staining

To determine the changes in neuromuscular junctions (NMJs), frozen sections of diaphragm muscles from both CFA and EAMG rats were prepared at a thickness of 7 μm for immunofluorescence. The muscle sections were initially blocked with 5% horse serum (ZSGB-Bio, China) at room temperature (RT) for 2 h. Subsequently, the sections were incubated overnight at 4 °C with tetramethylrhodamine-labeled α-BTX at a dilution of 1:400 in 5% horse serum. Following this incubation, the sections were treated with diamidino-2-phenylindole (DAPI) at a dilution of 1:2000 in PBS for 2 min at RT. Finally, the sections were observed and photographed using a confocal microscope (Zeiss, Germany).

### Immunohistochemistry

Forty-micrometer-thick frozen sections of the midbrain from both CFA and EAMG rats were obtained to determine the tyrosine hydroxylase (TH) changes by immunohistochemistry analysis. The midbrain sections were initially blocked with 5% horse serum (ZSGB-Bio, China) for 2 h. Subsequently, endogenous peroxidases were blocked by treating the sections with 0.3% H_2_O_2_ at RT for 10 min. To facilitate membrane ruptures, the sections were then incubated in 0.3% Triton X-100 for 15 min to obtain membrane ruptures. Sections were washed and subsequently stained with anti-TH antibody at a dilution of 1:400 in 5% horse serum, with overnight incubation at 4 °C. This was followed by a second incubation with horseradish peroxidase (HRP)-conjugated goat anti-rabbit IgG at a dilution of 1:1000 in PBS for 30 min at 37 °C. After washing, the substrate reaction was carried out at RT. Images were acquired using an optical microscope (Olympus, Japan).

### Enzyme-linked immunosorbent assay (ELISA)

The titers of AChRα subunit (R-AChR_97-116_)-specific antibodies and dopamine in serum and supernatant were determined by ELISA. Briefly, 96-well Nunc-Immuno MaxiSorp plates (Thermo Fisher Scientific) were coated with purified R-AChR_97-116_ in 100 μL of 0.1 M carbonate bicarbonate buffer (pH = 9.6) at a final concentration of 2 µg/ml at 4 °C overnight, followed by 2 h of blocking with 5% FCS at RT. Then, we added and incubated 100 μL of diluted serum (1:2000) or supernatant at RT for an additional 2 h. Next, rabbit anti-rat IgG antibody (Sigma, America) was added and incubated for an additional 2 h at RT. After thorough washing with PBS-Tween, goat anti-rabbit IgG (ZSGB-Bio, China) diluted at 1:2000 was added and incubated at 37 °C for 30 min. The color reaction was initiated with TMB substrate solution (eBioscience, America), and the reaction was halted by 2 mol/L H_2_SO_4_. The absorbance values were measured at 450 nm on an ELISA plate reader (Bio-Rad Laboratories, Inc. Hercules, CA). Commercial kits from ENZO (ENZO Life Science, America) were employed for the detection of total serum DA levels.

### CCK-8 analysis

Splenocytes derived from EAMG rats or bEnd.3 cells were seeded into 96-well plates with varying DA concentrations for a 48-h incubation period. Each well was seeded with 2 × 10^5^ splenocytes or 1.5 × 10^3^ bEnd.3 cells in a total volume of 200 μL. Subsequently, 10 μL of Cell Counting Kit-8 (CCK-8, Dojindo, Japan) reagent was added to each well. The incubation time for CCK-8 varied depending on the cell types: an additional 4 h for splenocytes, and 30–60 min for bEnd.3 cells. The absorbance values were measured at 450 nm on an ELISA plate reader (Bio-Rad Laboratories, Inc. Hercules, CA).

### Protein extraction and western blot

Protein samples were prepared by lysing total brain tissues or cells in cell lysis buffer (Beyotime), supplemented with phosphatase inhibitor cocktail A (Beyotime) and protease inhibitor cocktail phenylmethylsulfonyl fluoride. The protein samples were then normalized and denatured at 100 °C for 6–8 min and separated by 10% SDS–polyacrylamide gel electrophoresis. Then, the protein was transferred onto PVDF membranes (Millipore, Germany) via wet transfer. Membranes were blocked with 5% nonfat milk at RT for 3 h. Primary antibodies were diluted in Tris-buffered saline with 0.1% Tween 20 overnight at 4 °C. Various primary antibodies were used, including GAPDH (1:2000, ORIGENE), β-actin (1:2000, ORIGENE), TH (1:1000, CST), CHGB (1:2000, GeneTex), ZO1 (1:500, Thermo), ZO2 (1:200, Santa), CD31 (1:1000, Santa), and Non-P-Active-β-catenin (1:2000, ABclonal). The other antibodies against Occludin, Claudin5, CXC43, Wnt3a, GSK3β/p-GSK3β (Ser9), and β-catenin (1:500) were purchased from Wanlei Bio Co., Ltd. (Shenyang, China). The protein bands were detected by probing the blots with HRP phosphatase-conjugated secondary antibodies (goat-anti-mouse/rabbit, ZSGB-BIO) for 2 h at RT.

### Total RNA extraction and quantitative PCR

Total RNA was extracted from enriched CD4^+^ T cells, B220^+^ B cells, lymphocytes, the bEnd.3 cell line, and cerebral microvascular, midbrain tissues using TRIzol reagent (﻿Takara, USA) following the manufacturer’s instructions. Synthesis of cDNA was performed using the Takara cDNA synthesis kit. Quantitative PCR (qPCR) was conducted to assess differences in specific mRNA levels. The primer sequences for rats and mice (listed 5′–3′) used in this study are listed in Additional file 1: Table S2, S3. All samples were run in triplicate using the StepOne Plus PCR System (ThermoFisher Scientific, America).

### Flow cytometry

Single-cell suspensions prepared from spleens were stained with fluorochrome-conjugated antibodies against rat CD4 (PerCP), CD45R (PE), IL-17A (PE), Foxp3 (APC), CD80 (PE), CD86 (PE), MHC II (PE), CD45R (FITC), CD138 (PE) and CD69 (PE) purchased from eBioscience, BioLegend, R&D Systems, Thermo and Santa. For intracellular cytokine detection, splenocytes subjected to various treatments were collected and stimulated for 4–6 h with phorbol-12-myristate-13-acetate (50 ng/ml, Sigma-Aldrich) and ionomycin (1 µg/ml, Enzo) in the presence of brefeldin A (eBioscience, San Diego, CA). After permeabilization using Cytofix/Cytoperm (BD Biosciences, San Jose, CA), cells were stained with the appropriate antibodies. Data acquisition was performed using the BD FACSVerse flow cytometer and analyzed with FlowJo software (Tree Star).

### Statistical analysis

All experiments were conducted with three replicates or more. Statistical comparisons between two groups were analyzed using the Student’s *t* tests, and one-way or two-way ANOVAs were employed for data analysis involving more than two groups. GraphPad Prism 9 (GraphPad Software, San Diego, CA) was used for these statistical analyses. All data are presented as the mean ± standard deviation. A significance level of *P* < 0.05 was considered statistically significant (*P < 0.05; **P < 0.01; ***P < 0.001). The complete table consisting of all statistics, *p* values, and effect sizes (adjusted *R*^2^) can be found in Additional file 1: Table S4.

## Results

### Establishment of the EAMG animal model

**Fig. 1:**
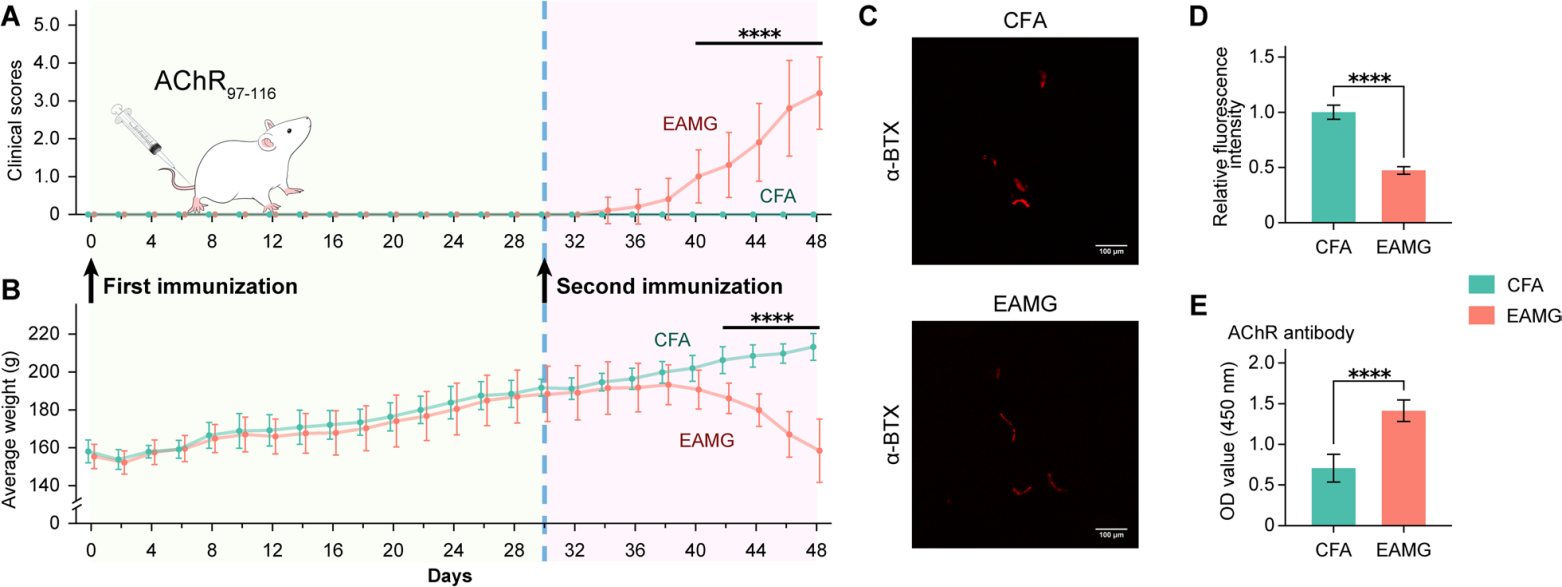
Establishment of the EAMG rat model. **A** Clinical scores and **B** body weight of 20 rats were assessed to evaluate the establishment of the rat model. Arrows and colors indicate the timing of immunizations on day 0 and day 30. Significant differences in clinical scores were observed on day 40, while significant differences in average weights were observed on day 42 using Sidak’s correction. **C** α-BTX staining and its fluorescence intensity (normalized to CFA) at a magnification of 100 μm. **D** The relative intensity in the CFA group is significantly higher than in the EAMG group. **E** The level of AChR-specific antibodies in serum was significantly higher in the EAMG group compared to the CFA group. Statistical analysis was performed using repeated measures ANOVA and *t* test, *P < 0.05, **P < 0.01, ***P < 0.001, ****P < 0.0001, *ns =* no significant difference

Rats were monitored and weighed every other day with blind to operators, starting from the day of the first immunization (day 0). As shown in Fig. 1A, the clinical scores of the EAMG group gradually increased from day 34 and exhibited a significant increase from day 40 compared to the CFA group (Fig. 1A). Body weight serves as an additional indicator of disease development when compared to the CFA group. Starting on the twelfth day after the second immune boost, the EAMG rats exhibited a significant decline in body weight (Fig. 1B). After the clinical score reached two points, the rats were randomly selected for the subsequent experiment.

To assess the alteration of AChR clusters in the NMJ, the diaphragm muscle was dissected and stained with α-BTX. The results clearly demonstrated a significantly decreased fluorescence intensity of α-BTX staining at the NMJ in the muscle section of the EAMG group, along with an apparent intermittent morphology (Fig. 1C, D, red), indicating damage and lost AChRs clustered at the NMJ. ELISA data showed a significant increase in serum anti-AChR antibody levels in EAMG rats (Fig. 1E). Collectively, these results indicate the successful establishment of the EAMG rat model.

### Hyperfunction of dopaminergic system in the midbrain of EAMG rats

**Fig. 2:**
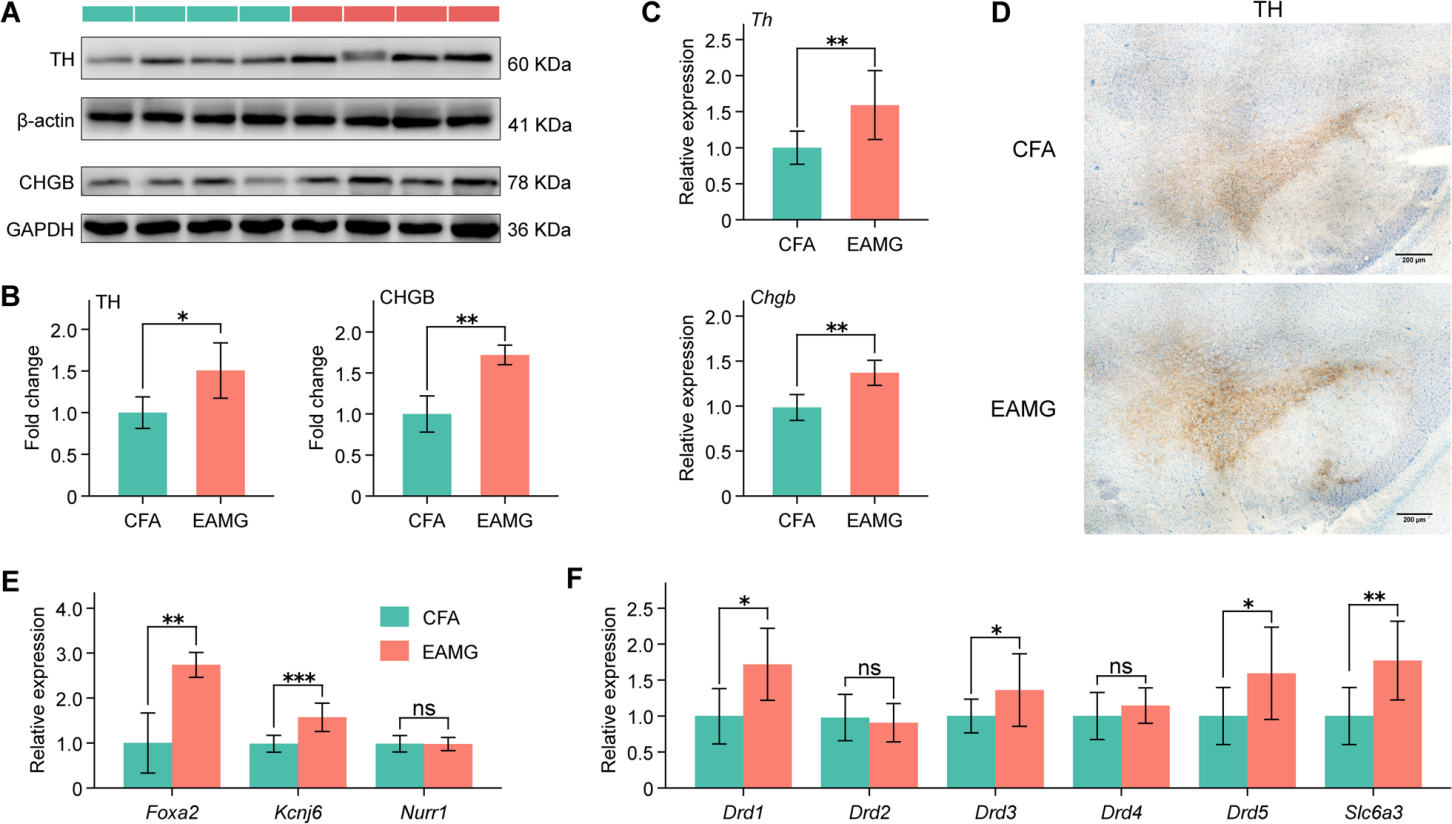
Hyperfunction of the dopaminergic system in the midbrain of EAMG rats. **A** The protein expression levels of TH and CHGB in the midbrain were compared between the CFA and EAMG groups using western blot. **B** TH and CHGB protein expression levels in the midbrain were significantly higher in the EAMG group. **C** The mRNA expression levels of *Th* and *Chgb* in the midbrain were significantly elevated in the EAMG group. **D** Representative immunohistochemistry staining of TH in the midbrain was performed at 200 μm magnification. **E** Higher expression levels of the transcription factor *Foxa2* and *Kcnj6* were also detected in the midbrain. No significant difference was observed in the expression of the *Nurr1* gene. **F** Gene expression of *Drd1*-*5* and *Slc6a3* genes in the midbrain revealed that *Drd1*, *Drd3*, *Drd5*, and *Slc6a3* showed significantly higher levels in the EAMG group, while *Drd2* and *Drd4* did not show the significant difference. Statistical analysis was performed using *t* tests, *P < 0.05, **P < 0.01, ***P < 0.001, ****P < 0.0001, *ns* = no significant difference

Previous studies have primarily focused on the peripheral immune regulation mechanism of MG while neglecting the alteration and regulatory effects of the CNS in MG. Therefore, it is crucial to direct more attention toward the CNS in MG. In our study, we unexpectedly discovered hyperfunction of dopaminergic neurons in the CNS of symptomatic EAMG rats. After separating the midbrain tissues containing the substantia nigra, we conducted western blot and qPCR analyses on total protein and RNA. Our results revealed significantly higher levels of TH (*Th*, a marker of dopaminergic neurons) and CHGB (*Chgb*, chromogranin B, a marker of CA-containing vesicles in dopaminergic neurons) proteins and their mRNAs in the EAMG group than in the CFA group (Fig. 2A–C). Additionally, immunohistochemistry experiments confirmed similar findings in independent samples (Fig. 2D). Abnormal upregulation of markers for dopaminergic neurons, such as *Foxa2* and *Kcnj6*, was observed in the midbrain of EAMG rats, indicating hyperactivity of dopaminergic neurons (Fig. 2E). Furthermore, compared to the control group, the mRNA levels of DA receptors (*Drd1*, *Drd3*, and *Drd5*) and the DA transporter (*Scl6a3*) significantly increased in the midbrain of experimental rats. However, the expression of DA receptors (*Drd2* and *Drd4*) remained statistically unaltered between groups (Fig. 2F). These findings collectively demonstrated hyperfunction of the dopaminergic system in the midbrains of EAMG rats.

### Gene expression screening and pathway enrichment by proteomic analysis

**Fig. 3:**
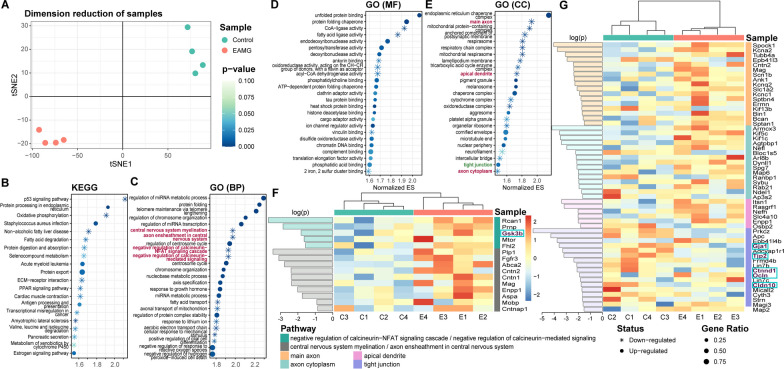
Visualization of differentially expressed genes (DEGs) in eight pathways. **A** Two-dimensional t-SNE plot showing the separation between CFA and EAMG rats, with each color representing a different sample group. **B**–**E** The top enriched pathways with the highest NES were plotted in each subplot for sequenced proteins in the Kyoto Encyclopedia of Genes and Genomes (KEGG, **B**) and the biological process (BP, **C**), molecular function (MF; **D**), and cellular component (CC, **E**) components in the GO database, respectively. The CNS-related pathways are highlighted in dark crimson, and one pathway associated with the epithelium is highlighted in dark cyan. **F**–**G** Two heatmaps illustrate the levels of differentially expressed genes within eight pathways. In each heatmap, the left panel shows the log-transformed *p* values obtained by the *t* tests, while the central panel shows the relative expression value normalized using the *z* scores

Proteomic characterization of human brain tissue is increasingly used to identify potential novel biomarkers and drug targets for diverse neurological diseases. In this study, total brain tissues were extracted from CFA and EAMG rats receiving two doses of AChR immunization. The two groups of deskinned brain tissues were sent to Jingjie PTM BioLab (Hangzhou) Co., Ltd for mass spectrometry analysis. As depicted in Fig. 3A, t-SNE analysis effectively distinguished the disease and normal samples. Subsequently, we performed GSEA using complete expression data in the KEGG database (Fig. 3B) and GO database (Fig. 3C–E) to gain further insights into the disrupted signaling pathways in EAMG rats. In the GO database, we examined three datasets, namely Biological Process (BP; Fig. 3C), Molecular Function (MF; Fig. 3D), and Cellular Component (CC; Fig. 3E), each generating an enrichment plot. The enrichment results from the GO database revealed seven CNS-specific pathways (highlighted in dark crimson) and one epithelium-related pathway (highlighted in middle green), suggesting the presence of CNS lesions in EAMG rats. Additionally, we drew expression heatmaps (Fig. 3F–G) and found 80 differentially expressed genes within these eight pathways. From the heatmap, we selected six genes (*Gsk3b*, *Gja1*, *Tjp2*, *Ctnnd1*, *Ocln*, and *Cldn10*) and hypothesized that both the cerebral microvasculature and TJs were damaged in EAMG rats.

### The tight and adherence junctions of cerebral microvasculature in EAMG rats

**Fig. 4:**
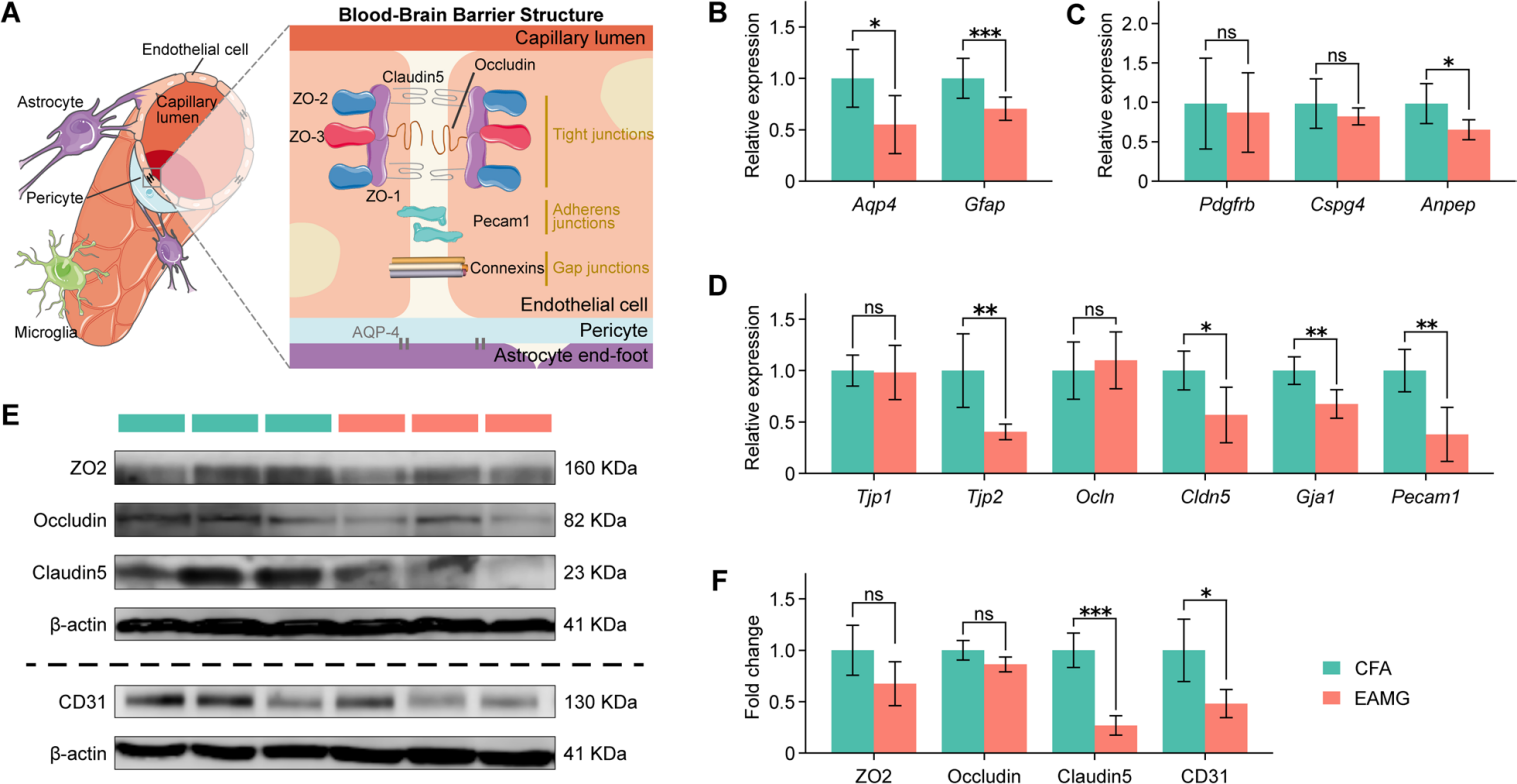
Changes in structural molecules in the cerebral microvasculature in EAMG rats. **A** The diagram represents the structure of the blood–brain barrier. **B** The relative mRNA levels of *Aqp4* and *Gfap* in the cerebral microvascular tissue were significantly higher in CFA rats compared to EAMG rats. **C** The relative mRNA levels of *Anpep* were significantly higher in CFA rats than in EAMG rats, whereas no statistical difference was observed for *Pdgfrb* and *Cspg4* in cerebral microvascular using qPCR. **D** Among the tested gene sets in cerebral microvascular tissues (*Tjp1*, *Tjp2*, *Ocln*, *Cldn5*, *Gja1*, and *Pecam1*), CFA rats exhibited significantly higher levels except for *Tjp1* and *Ocln*. **E** The protein expression levels of ZO2, Occludin, Claudin5, and CD31 in cerebral microvascular tissues were compared between the CFA and EAMG groups using western blot. **F** The expression levels of Claudin5 and CD31 in the cerebral microvascular tissues were significantly higher in the CFA group, while ZO2 and Occludin exhibited no significant differences. Statistical analysis was performed using *t* tests, *P < 0.05, **P < 0.01, ***P < 0.001, ****P < 0.0001, *ns* = no significant difference

The main proteins in EC-cell junctions are presented in Fig. 4A. To assess the altered status of these junction proteins in EAMG rats, the cerebral microvasculature was successfully isolated and purified. The high purity of the isolation was confirmed via HE staining and qPCR analysis, revealing the tubular structure and the absence of neuronal markers (*Syp* and *Tubb3* genes) (Additional file 1: Fig. S1A–C). Subsequently, the content of BBB constituent cells (ECs, PCs, and glial cells) was examined by qPCR between groups. In the EAMG group, the expression of two astrocyte markers, the glial water channel aquaporin 4 (*Aqp4*) and the glial fibrillary acidic protein (*Gfap*) significantly decreased (Fig. 4B), while the PC genes (platelet-derived growth factor receptor beta (*Pdgfrb*) and chondroitin sulfate proteoglycan 4 (*Cspg4*)) remained unaltered except for a decrease in the alanine aminopeptidase (*Anpep*) gene (Fig. 4C). Moreover, the expression levels of EC markers, including *Tjp2*, *Cldn5*, *Gja1* and *Pecam1*, were downregulated in EAMG rats compared to the CFA group, while the expression of the remaining genes showed no distinct alterations (Fig. 4D). Further confirmation of the expression of TJ and AJ proteins in ECs was obtained via western blot analysis, which revealed a decline in Claudin5 (TJ) and CD31 (AJ) expression in EAMG rats compared to the control group, consistent with the qPCR results (Fig. 4E, F). Overall, these findings align with the proteomic analysis and confirm the reduced expression of tight (Claudin5, *Cldn5*) and adherence (CD31, *Pecam1*) junction molecules in cerebral vascular ECs in the EAMG group. However, no significant BBB leakage was observed upon the intravenous injection of cadaverine in EAMG rats, indicating no functional impairment of the BBB, which may be attributed to the short-term cycles of the EAMG rats (Additional file 1: Fig. S2).

### Dopamine inhibited the levels of tight and adherence junction proteins in endothelial cells

**Fig. 5:**
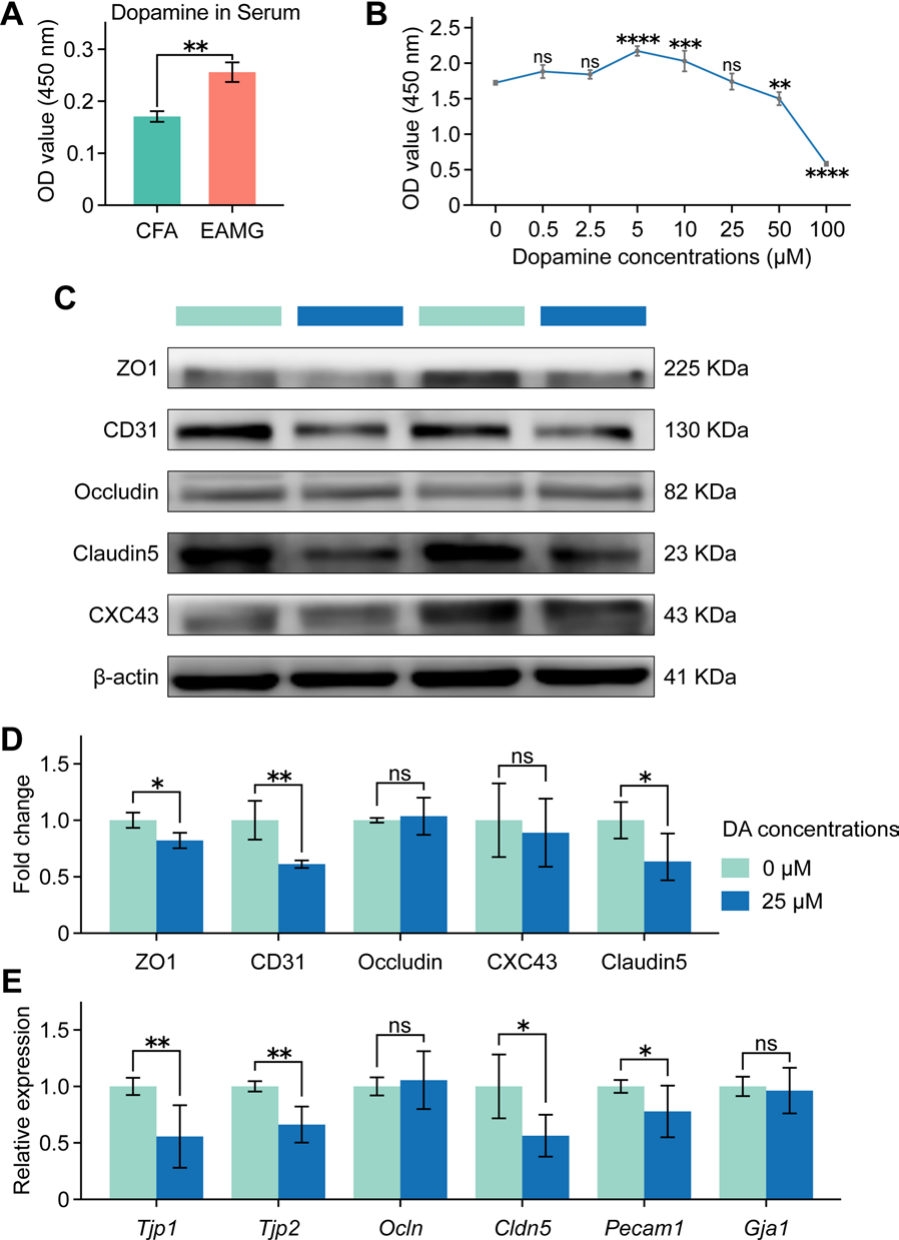
The impact of dopamine incubation in the periphery on the bEnd.3 in vitro was investigated. **A** The dopamine level in serum was significantly higher in EAMG rats, as measured by ELISA. **B** The proliferative effects of DA on the bEnd.3 cell lines by CCK-8. **C** The expression of ZO1, CD31, Occludin, Claudin5 and CXC43 proteins in bEnd.3 cell lines was compared after incubation by western blot. **D** The dopamine-treated bEnd.3 cell lines exhibited significantly lower levels of ZO1, CD31, and Claudin5 expression. **E** Among the tested gene sets in serum (*Tjp1*, *Tjp2*, *Ocln*, *Cldn5*, *Pecam1*, and *Gja1*), dopamine-treated cell lines exhibited significantly reduced levels of dopamine, except for *Ocln* and *Gja1* using qPCR. Statistical analysis was performed using one-way ANOVA and *t* tests, *P < 0.05, **P < 0.01, ***P < 0.001, ****P < 0.0001, *ns* = no significant difference

Consistent with the hyperfunction of the dopaminergic system in the CNS, an elevated DA level was observed in the serum of EAMG rats compared to the CFA group (Fig. 5A). Previous studies have demonstrated that increased peripheral DA impairs angiogenesis by inhibiting angiotensin receptor type 1 expression in ECs during post-ischemic healing disease [34]. To verify whether the abovementioned impairment of endothelial junctions was caused by elevated DA levels in both the periphery and CNS, bEnd.3 cells were incubated for 48 h with various concentrations of DA hydrochloride. Data showed that 25 μmol/L DA did not proliferate bEnd.3 cells (Fig. 5B), it significantly reduced the expression of ZO1, Claudin5, and CD31 at both the protein and gene levels (Fig. 5C–E). These findings indicated that DA could inhibit the expression of TJ and AJ proteins in bEnd.3 cells, which is essential to maintain the BBB structural integrity.

### Dopamine impairs endothelial cells via the Wnt/β-catenin pathway

**Fig. 6:**
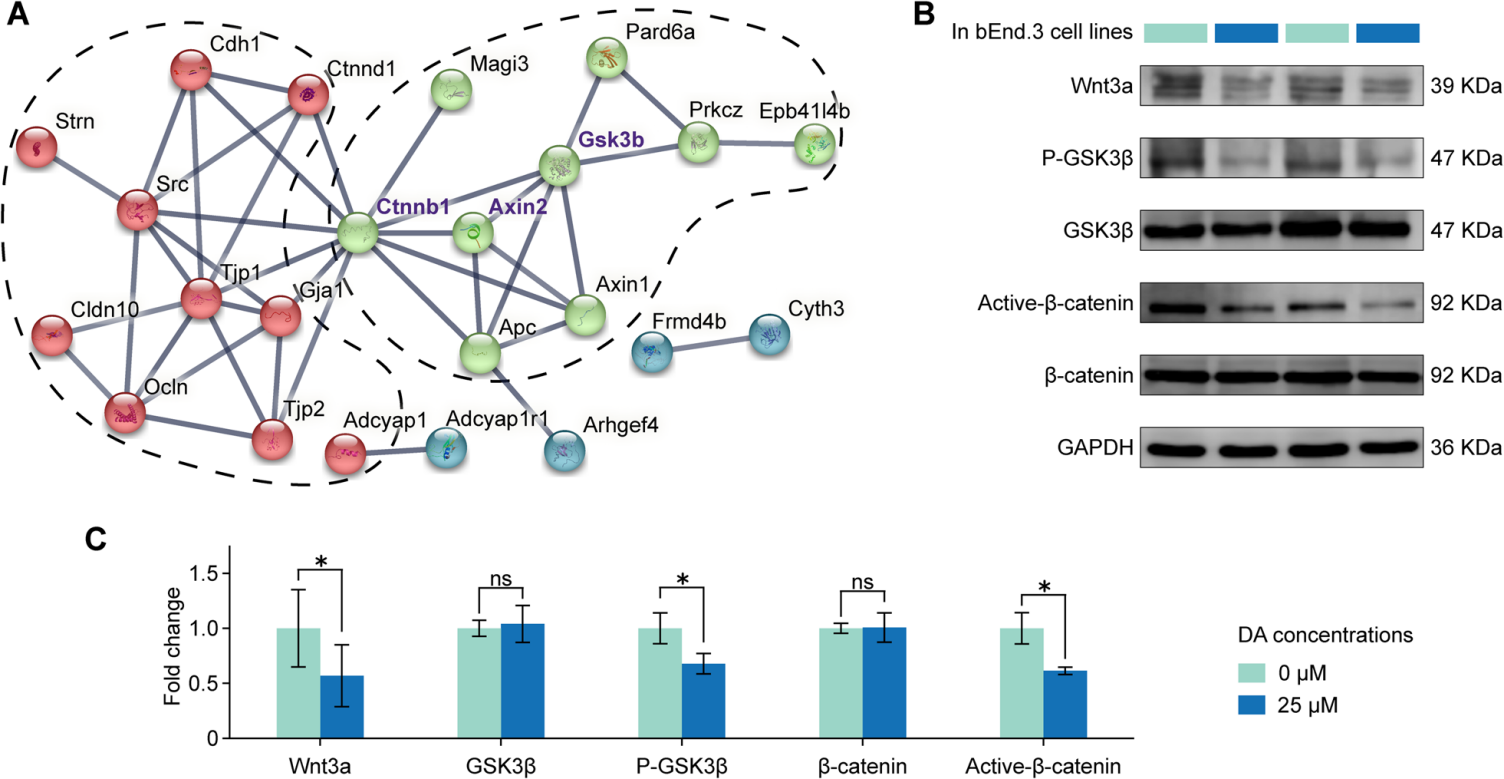
Dopamine disrupts endothelial junctions through the Wnt/β-catenin signaling pathway. **A** The protein–protein interaction subnetwork was to visualize the interactions between tight junction proteins. K-means clustering was applied to color the genes in the graph and divide the subnetwork into two main clusters (highlighted in red and green) and a secondary cluster (highlighted in blue). Genes with high connectivity were selected for the following analysis, including *Ctnnb1*, *Axin2*, and *Gsk3b*. **B** The protein expression levels of Wnt3a, GSK3β/p-GSK3β, and β-catenin/active-β-catenin in the bEnd.3 cell lines were compared between without dopamine and with dopamine-treated groups using western blot. **C** The protein expression levels of Wnt3a, GSK3β/p-GSK3β, and β-catenin/active-β-catenin in the bEnd.3 cell lines were compared using western blot, revealing lower levels of Wnt3a, p-GSK3β, and active-β-catenin in dopamine-treated cell lines. Statistical analysis was performed using *t* tests, *P < 0.05, **P < 0.01, ***P < 0.001, ****P < 0.0001, *ns* = no significant difference

To comprehensively understand the mechanisms underlying TJ and AJ alterations, we examined differentially expressed genes within the protein–protein interaction (PPI) network, focusing on those associated with TJs and AJs in the STRING database (https://string-db.org). Upon inputting all the DEGs into the STRING database, we extracted a small subnetwork. Following K-means clustering, we extracted two main clusters (marked as red and green) and a secondary cluster (marked as navy blue). Notably, all the genes we focused on (i.e., *Tjp1*, *Tjp2*, *Ocln*, *Gja1*, and *Cldn10*) were part of the red cluster. Among the two main clusters, *Ctnnb1* was the bridge node. In Fig. 6A, *Axin2* and *GSK3b* connected to *Ctnnb1* within the green cluster, and they were considered key molecules in the classical Wnt signaling pathway. Based on these findings, we postulated that DA might induce EC impairment via the Wnt/β-catenin pathway.

Several studies have also reported that restoring the expression of CD31 and Claudin5 can enhance BBB integrity through the Wnt/β-catenin signaling pathway [22, 42]. Hence, we investigated whether DA affected ECs through the Wnt/β-catenin signaling pathway. Therefore, we treated bEnd.3 cells with DA and analyzed the protein levels of Wnt3a, total GSK3β/p-GSK3β, and total β-catenin/active-β-catenin by western blotting. As illustrated in Fig. 6B, C, 25 μmol/L DA significantly reduced the expression of Wnt3a, p-GSK3β, and active-β-catenin in bEnd.3 cells when compared to the nontreated control. However, DA had no significant effect on GSK3β and total β-catenin protein levels (Fig. 6B, C). Based on the findings from Figs. 5, 6, we conclude that DA inhibits the expression of TJ and AJ proteins in bEnd.3 cells by decreasing the expression of proteins associated with the Wnt/β-catenin pathway. The bEnd.3 cells were stimulated with DA (25 μM) at a concentration of 10 μM, along with 10 μM of SCH23390 (dopamine D1 receptor antagonist) and haloperidol (dopamine D2 receptor antagonist). Compared to DA treatment group, both cell lines treated with SCH23390 and haloperidol resulted in increased expression levels of CD31, p-GSK3β and active-β-catenin. However, they did not induce statistically significant changes in Cluadin5 and Wnt3a levels, despite a noticeable increasing trend observed over 48 h in vitro (Additional file 1: Fig. S4).

### The effects of dopamine on AChR-specific Th cells and B cells

**Fig. 7:**
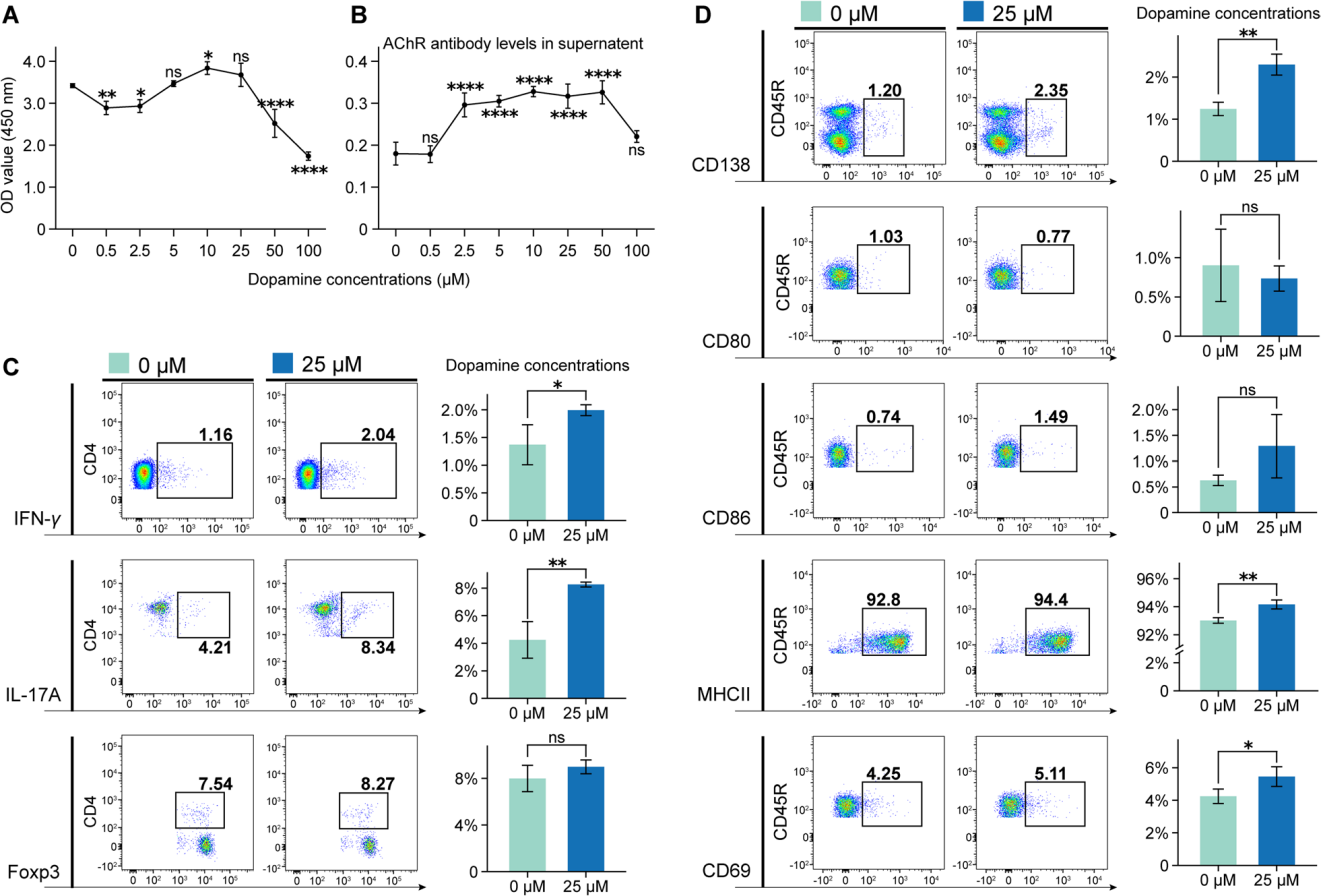
The effects of dopamine on T and B cells. **A** The proliferative effects of dopamine on lymphocytes by CCK-8. **B** AChR-specific antibody in the supernatant were measured using ELISA. Statistical analysis was performed using one-way ANOVA in Panel A and B, multiple comparisons were performed using Dunnett’s test with the DA concentration of 0 μmol/L. **C** The incubation of dopamine significantly increased the percentage of Th1 and Th17 cells, while the proportion of Treg cells remained unchanged. **D** Incubated dopamine led to a significant increase in the percentage of CD138, MHC II^+^ and CD69^+^ B cells, while the proportions of CD80 and CD86 B cells remained unchanged. Statistical analysis was performed using *t* tests in Panel C and D, *P < 0.05, **P < 0.01, ***P < 0.001, ****P < 0.0001, ns = no significant difference

As mentioned above, MG is a classical neurological autoimmune disease characterized by overactivated immune cells. Additionally, DA was also verified to have immune-regulatory effects through its receptors and transporters (Additional file 1: Fig. S3A, B). The inflammatory response is promoted by Th1 (IFN-γ-secreting T cells) and Th17 (IL-17A-secreting T cells) cells through cytokine secretion, while Tregs (Foxp3-positive T cells) suppress inflammation by upregulating immunosuppressive molecules, tissue homing receptors, and repressing genes. Regarding B cell markers, CD80, CD86, and MHC II serve as antigen-presenting molecules, CD69 indicates cell activation, and CD138 represents a core plasma cell marker. To investigate the role of elevated peripheral DA on EAMG-specific immune cells, single-cell suspensions of EAMG splenocytes were stimulated with AChR (10 μg/mL) and different concentrations of DA (0, 0.5, 2.5, 5, 10, 25, 50, and 100 μmol/L). Our results showed that DA at concentrations of 5 and 25 μmol/L did not affect the proliferation of splenocytes (Fig. 7A), but stimulated AChR-antibody secretion by B cells at almost all other concentrations except for 0.5 and 100 μmol/L (Fig. 7B). Considering the proliferative effects observed on bEnd.3 cells validated in Fig. 5B, we selected a concentration of 25 μmol/L of DA for the subsequent 48 h of in vitro incubation with splenocytes.

The incubation of DA significantly increased the percentage of Th1 and Th17 cells, while the proportion of Treg cells remained unchanged (Fig. 7C). Besides, 25 μmol/L DA notably enhanced the percentages of CD138, MHC II, and CD69 expression in B cells (Fig. 7D), but had little effect on the CD80 and CD86 ratios (Fig. 7D). These findings suggested that due to the involvement of immune cells in the occurrence and development of EAMG [43–45], the abnormal activation of Th and B cells by DA could aggravate the progression of EAMG.

### The influence of activated AChR-specific-immune cells on endothelial cells

**Fig. 8:**
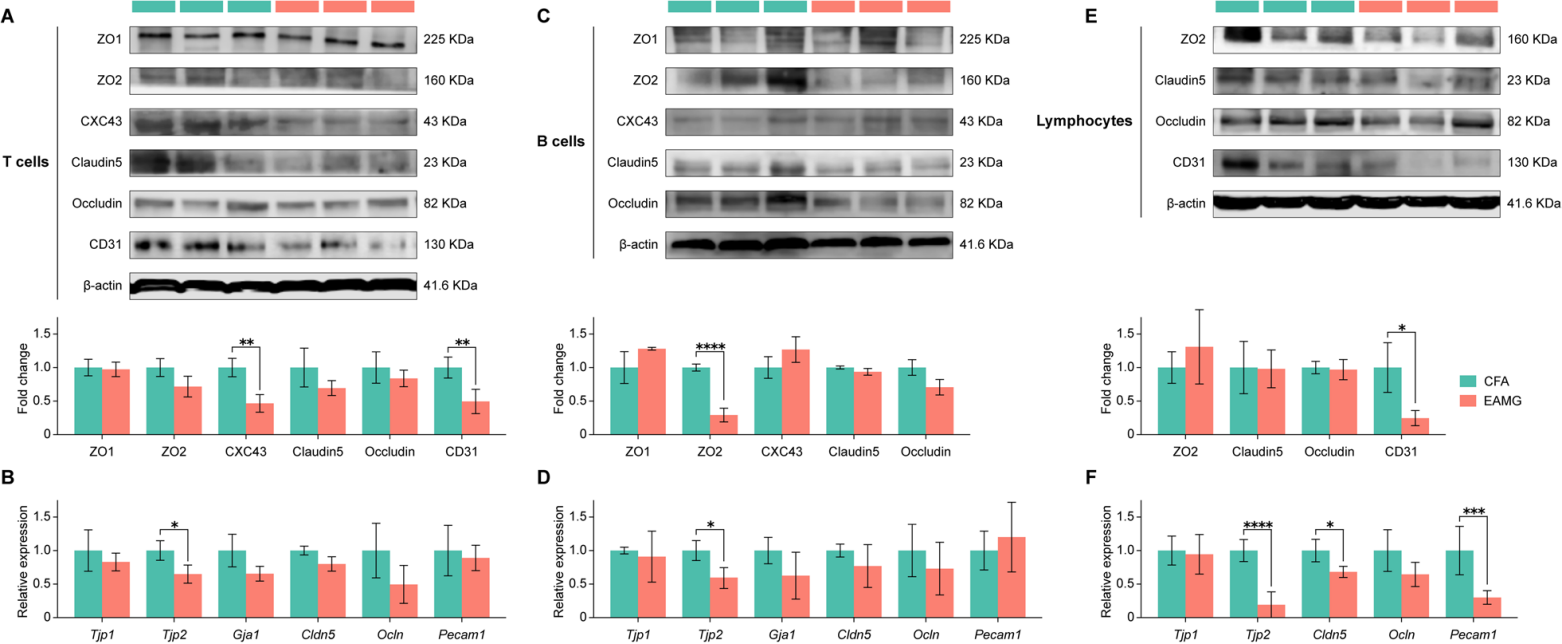
The influence of disease-specific immune cells on endothelial cells. The expressions of ZO1, ZO2, CXC43, Claudin5, Occludin, and CD31 in endothelial cells cocultured with T cells (**A**, **B**), B cells (**C**, **D**), and lymphocytes (**E**, **F**) from both the CFA and EAMG groups was analyzed using western blotting and qPCR. Statistical analysis was performed using *t* tests, *P < 0.05, **P < 0.01, ***P < 0.001, ****P < 0.0001, *ns* = no significant difference

In addition to the direct effects of DA on ECs, we also assessed how DA indirectly influences ECs through DA-activated immune cells. Our findings revealed an elevation of DA in the peripheral circulation of EAMG rats (Fig. 5B) and showed that high DA incubation had a proinflammatory effect on AChR-specific T and B cells (Fig. 7B–D), indicating the lymphocytes in the peripheral immune organs of EAMG rats were activated by DA and AChR. Consequently, bEnd.3 cells were cocultured with lymphocytes, enriched CD4^+^ T cells, or B220^+^ B cells from two animal groups for 48 h before harvesting. Results presented in Fig. 8 demonstrate that EAMG-derived T cells inhibited the protein expression of CXC43 and CD31 in bEnd.3 cells compared to CFA-derived T cells (Fig. 8A). Additionally, EAMG-derived T cells suppressed the mRNA expression of *Tjp2* in bEnd.3 cells (Fig. 8B). The protein and RNA levels of ZO2 (*Tjp2*) in bEnd.3 cells were significantly reduced by EAMG-derived B cells (Fig. 8C, D). Furthermore, EAMG-derived lymphocytes suppressed the protein and mRNA expression of CD31 (*Pecam1*) and decreased the gene levels of *Tjp2* and *Cldn5* (Fig. 8E, F). In summary, immune cells activated by DA and AChR in EAMG rats caused structural impairment in the integrity of TJs and AJs in bEnd.3 cells.

## Discussion

Previous studies have explored the involvement of central neurological symptoms in some patients with MG. However, the related pathological changes in the CNS have not been investigated extensively. In this study, we provided novel evidence by demonstrating the pathological elevation of DA levels in both the CNS and peripheral circulation suppressed the expression of TJs and AJs in the cerebral microvascular ECs in the EAMG rat model via the Wnt/β-catenin signaling pathway. This disruption of the BBB microstructural integrity could potentially contribute to the CNS manifestations observed in some MG patients (see Graphical abstract).

Throughout the history of MG, the focus of research has predominantly been on the clinical symptoms of muscle weakness and fatigability mediated by pathological immune cells. However, some MG patients experience CNS symptoms in intermediate and advanced stages, including depression, anxiety disorders, headaches, dysautonomia, sleep disorders, and epilepsy [46, 47]. These CNS symptoms could be partially attributed to drug side effects (specifically, intravenous immunoglobulin, ravulizumab, and pyridostigmine) [48–50]. There are also cases where these symptoms arise from unknown factors, which require careful consideration. However, current research on CNS symptoms in MG has been limited to CSF analysis, clinical electroencephalograms, and neuropsychological tests. Reports have shown an increased proportion of CD4^+^ T cells in the CSF of MG patients [51]. Additionally, the IgG concentrations in the CSF of certain MG patients were significantly higher compared to healthy controls [12, 13]. Furthermore, abnormal electroencephalograph recordings indicated CNS disturbances in 14 out of 118 MG patients (8 diffuse slow abnormalities, 6 focal slow abnormalities) [11].

DA plays an integral regulatory role in the CNS, making the dopaminergic system relevant to various neurological disorders such as PD and schizophrenia [52, 53]. PD is characterized by motor symptoms including rigidity, resting tremor, bradykinesia, and the loss of dopaminergic neurons [28]. Schizophrenic patients exhibit elevated DA concentrations and receptor densities in several subcortical brain regions compared to healthy controls, contributing to anxiety and depression [54]. Lewis rats are used in the majority of contemporary MG models because they are thought to be a recognized representation of MG [38]. In our study, we provided novel evidence by confirming the abnormal elevation of DA levels and its receptors in the midbrain of EAMG rats at both the gene and protein levels (shown in Fig. 2). DA serves as a crucial immunomodulator, synthesized and produced by various immune cells, especially following activation [35–37]. Furthermore, MG patients exhibit lower serum melatonin levels [55] and deficit central cholinergic innervation when compared to healthy controls, leading to cognitive impairment [56]. This deficiency in melatonin or acetylcholine could potentially contribute to the elevated DA levels, as melatonin or acetylcholine is mutually balanced and restrained with DA [57–59]. This observation further strengthens the reliability of our findings regarding the increased DA levels in both the CNS and peripheral serum. Furthermore, we demonstrated that elevated DA levels mediated BBB microstructural integrity destruction by impairing TJs and adherence junctions (Fig. 5), a finding not previously reported in the literature. Considering the regulatory effects of DA on the CNS [60, 61], the abnormally increased DA in nuclei regions may provide insights into the involvement of central neurological symptoms in MG patients.

In addition to its regulatory effects on the CNS, DA also plays significant roles in the endothelial system, immune system, and other systems, including ECs [32], mast cells [34, 62], T cells [63], B cells [37, 64], and dendrite cells [65]. Given our observed CNS pathological alterations in EAMG rats, we hypothesized that the increased levels of DA in both the CNS and peripheral circulation might impact brain microvascular ECs. As depicted in Fig. 5, treatment with 25 μmol/L DA resulted in a significant decrease in the expression of proteins associated with TJs (ZO1, Claudin5) and adherence junctions (CD31) in ECs.

TJ and AJ are crucial components specific to ECs that play a key role in maintaining BBB integrity. Repression or damage to these junction barrier proteins can result in the disruption and increased permeability of the BBB [19, 20, 23, 24]. Although previous studies have indicated that the BBB remains intact and functionally unchanged in patients with MG [66, 67], our findings in Fig. 4 demonstrate a downregulation of Claudin5 (*Cldn5*), CXC43 (*Gja1*) and CD31 (*Pecam1*) expression in cerebral microvascular ECs of EAMG rats as assessed by western blot and qPCR. This downregulation suggests a possible breakdown and opening of the BBB in EAMG rats, which provides a solid basis for further investigation into CNS changes in both MG and EAMG.

To investigate differential gene expression in the entire PPI network, we performed K-means clustering and identified *Ctnnb1* and *Gsk3b* genes, which are the key molecules in the classical Wnt signaling pathway responsible for maintaining BBB integrity. Brain neovascularization involves several signaling pathways, including PI3K/AKT pathway, NF-κB pathway, and cAMP signaling pathway [30, 34, 68]. Among these pathways, the Wnt/β-catenin signaling pathway is known to play an important role in angiogenesis in the CNS [69, 70].

In the canonical Wnt pathway, the central coactivator β-catenin is maintained at low levels by a degradation complex comprising Axin, adenomatosis polyposis protein (APC), creatine kinase 1 (CK1α), and GSK3β, which phosphorylates β-catenin, leading to its ubiquitination and degradation. Consequently, it maintains low levels of β-catenin in the cytoplasm, preventing its entry into the nucleus for transcription initiation [21]. Activation of the Wnt pathway disrupts the stability of the degradation complex, allowing adequate β-catenin to accumulate in the cytoplasm and translocate into the nucleus. Once in the nucleus, β-catenin combines and interacts with TCF/LEF transcription factors to initiate the transcription of target genes [71, 72]. Therefore, we focused on the Wnt3a/GSK3β/β-catenin pathway. As depicted in Fig. 6, our in vitro experiments demonstrated that DA significantly impaired the expression of CD31 and Claudin5 in ECs via the activation of Wnt3a/GSK3β/β-catenin signaling.

As mentioned above, DA can promote or inhibit the activation, differentiation, and antibody secretion of immune cells [73, 74]. We also observed increased peripheral blood DA levels in EAMG rats. In vitro incubation with a concentration of 25 μmol/L of DA promoted Th1 and Th17 cell differentiation and B cell activation, as illustrated in Fig. 7. These findings suggested a mobilization of both T and B cells in vivo in EAMG rats due to the elevated DA levels in serum. We further investigated the impact of disease (AChR and DA)-specific inflammatory cells on ECs. Figure 8 illustrates that AChR-specific and DA-activated T and B cells suppressed the expression of TJ and AJ proteins, potentially compromising the integrity of the BBB structures maintained by ECs.

Although we verified the damaging effect of the cerebral microvasculature at both the gene and protein levels, the BBB fluorescence leakage assay (Additional file 1: Fig. S1) did not show significant disruption in BBB integrity. This apparent contradiction may be attributed to the short survival period (35–55 days) of EAMG rats, which may not fully reflect the long-term or lifelong impacts observed in MG clinical patients. Based on these findings, we hypothesize that long-term or lifelong effects of DA stimulation and AChR-specific lymphocyte activation in MG patients could invade brain microvascular ECs, causing irreversible damage to the TJ integrity of ECs and ultimately leading to the impairment of BBB microstructural integrity.

## Conclusions

In summary, our study revealed pathological alterations in the CNS of EAMG rats. Furthermore, we interpreted that elevated DA in both central and peripheral systems contributes to the impairment of cerebral microvascular ECs, which occurs through the activation of the Wnt/β-catenin pathway.

### Supplementary Information

**Additional file 1: Note S1.** Sample preparation. **Note S2.** Mass spectrometry analysis. **Note S3.** Bioinformatics tools and databases. **Figure S1.** Cerebral microvascular extraction and identification in CFA and EAMG rats. The cerebral microvascular was confirmed by the tubular structure via **A** optical microscope and **B** HE staining. **C** the absence of the neuronal markers (*Syp* and *Tubb3* genes) via qPCR. Statistical analysis was performed using *t* test, ***P < 0.001, ****P < 0.0001. **Figure S2. **The leakage of BBB in EAMG rats. The brain, spleen and kidney fluorescence, “ + ” and “−” indicated with or without Alexa flour 488 cadaverine injection (40 × magnification). **Figure S3.** The expression of *DRD1-DRD5* and *Scl6a3* genes in T cells (**A**) and B cells (**B**) of CFA and EAMG groups. Statistical analysis was conducted using *t* test, *P < 0.05, **P < 0.01, ns = no significance. **Figure S4**. The impact of SCH23390 (dopamine D1 receptor antagonist) and Haloperidol (dopamine D2 receptor antagonist) on bEnd.3 cells was investigated. **A**, **B** The proliferative effects of SCH23390 and Haloperidol on bEnd.3 cell line were assessed using CCK-8 assays. (C-D) The expression of CD31 was significantly increased upon incubation with both SCH23390 and Haloperidol, while Claudin5 remained unchanged. Additionally, the expression levels of Wnt3a in bEnd.3 cell line were examined through western blot, showing no significant changes in SCH23390 and Haloperidol treated cell lines. However, the results examined through western blot also showed elevated levels of p-GSK3β and active-β-catenin in SCH23390-treated cell line. Statistical analysis was conducted using one-way ANOVA, *P < 0.05, **P < 0.01, ***P < 0.001, ****P < 0.0001, ns = no significant difference. **Figure S5.** The effects of dopamine on T and B cells were examined. **A** Upon dopamine incubation, the mean fluorescence intensity (MFI) level of Th17 cells were significantly increased, whereas the MFI of Th1 and Treg cells exhibited no significant changes. **B** The dopamine exposure resulted in a significant increase in the MFI of CD86^+^ B cells and CD69^+^ B cells. Conversely, the MFI of CD80^+^ B cells decreased, and MHC II^+^ B cells remained unsignificant. Statistical analysis was conducted using *t* test, *P < 0.05, **P < 0.01, ns = no significant difference. **Figure S6.** Diaphragm samples were analyzed using immunofluorescence and HE staining. We performed **A** α-BTX and DAPI staining and **B** HE staining in the CFA group. We also performed **C** α-BTX and DAPI staining and **D** HE staining in the EAMG group. Blue staining represents DAPI, and α-BTX is the red staining highlighted by the white arrow. **Figure S7.** The effect of SCH23390 (dopamine D1 receptor antagonist) on EAMG rats was investigated by gavage every other day after the first immunization in vivo. **A** Clinical scores. **B** Survival probability and number at risk. **Table S1.** The criterion for each EAMG score is listed below. **Table S2.** The rat primer sequences are listed below. **Table S3.** The mouse primer sequences are listed below. **Table S4.** The detailed statistical results.

## Data Availability

The data in our study are available from the corresponding author upon reasonable request.

## Abbreviations

MG: Myasthenia gravis
EAMG: Experimental autoimmune myasthenia gravis
AChR: Acetylcholine receptor
Th cells: T helper cells
DA: Dopamine
α-BTX: Alpha-bungarotoxin
NMJ: Neuromuscular junction
CNS: Central nervous system
ECs: Endothelial cells
PCs: Pericytes
MS: Multiple sclerosis
PD: Parkinson’s disease
TJ: Tight junctions
AJ: Adherence junctions
