# Supplementary material for: Impaired cerebral microvascular endothelial cells integrity due to elevated dopamine in myasthenic model
Source: J Neuroinflammation. 2024 Jan 4;21:10. doi: 10.1186/s12974-023-03005-3 (PMC10765813; doi:10.1186/s12974-023-03005-3)
Supplement: Supplementary file 1 — Additional file 1: Note S1. Sample preparation. Note S2. Mass spectrometry analysis. Note S3. Bioinformatics tools and databases. Figure S1. Cerebral microvascular extraction and identification in CFA and EAMG rats. The cerebral microvascular was confirmed by the tubular structure via A optical microscope and B HE staining. C the absence of the neuronal markers (Syp and Tubb3 genes) via qPCR. Statistical analysis was performed using t test, ***P < 0.001, ****P < 0.0001. Figure S2. The leakage of BBB in EAMG rats. The brain, spleen and kidney fluorescence, “ + ” and “−” indicated with or without Alexa flour 488 cadaverine injection (40 × magnification). Figure S3. The expression of DRD1-DRD5 and Scl6a3 genes in T cells (A) and B cells (B) of CFA and EAMG groups. Statistical analysis was conducted using t test, *P < 0.05, **P < 0.01, ns = no significance. Figure S4. The impact of SCH23390 (dopamine D1 receptor antagonist) and Haloperidol (dopamine D2 receptor antagonist) on bEnd.3 cells was investigated. A, B The proliferative effects of SCH23390 and Haloperidol on bEnd.3 cell line were assessed using CCK-8 assays. (C-D) The expression of CD31 was significantly increased upon incubation with both SCH23390 and Haloperidol, while Claudin5 remained unchanged. Additionally, the expression levels of Wnt3a in bEnd.3 cell line were examined through western blot, showing no significant changes in SCH23390 and Haloperidol treated cell lines. However, the results examined through western blot also showed elevated levels of p-GSK3β and active-β-catenin in SCH23390-treated cell line. Statistical analysis was conducted using one-way ANOVA, *P < 0.05, **P < 0.01, ***P < 0.001, ****P < 0.0001, ns = no significant difference. Figure S5. The effects of dopamine on T and B cells were examined. A Upon dopamine incubation, the mean fluorescence intensity (MFI) level of Th17 cells were significantly increased, whereas the MFI of Th1 and Treg cells exhibited no significant changes. B The d [file 12974_2023_3005_MOESM1_ESM.pdf]

Supplementary Materials

Table of Contents

Supplementary Note 1: Sample Preparation 1
Protein extraction 1
Digestion 1
TMT labeling........ 1
HPLC fractionation 1
Supplementary Note 2: Mass Spectrometry Analysis 2
Mass spectrometer 2
Analysis 2
Database searching 2
Supplementary Note 3: Bioinformatics tools and databases 3
Gene Ontology (GO) database 3
KEGG PATHWAY 3
Gene set enrichment analysis (GSEA) 4
Protein-protein interaction and STRING database 4
t-distributed Stochastic Neighbor Embedding (t-SNE) 5
Supplementary Figure 1 7
Supplementary Figure 2 8
Supplementary Figure 3 9
Supplementary Figure 4 10
Supplementary Figure 5 11
Supplementary Figure 6 12
Supplementary Figure 7 13
Supplementary Table 1 14
Supplementary Table 2 15
Supplementary Table 3 16
Supplementary Table 4 17
Supplementary Table 5 21

Supplementary Note 1: Sample Preparation

Protein extraction

The sample was ground into cell powder using liquid nitrogen and then transferred to a 5-mL centrifuge tube. Then, four volumes of lysis buffer (8 M urea, 1% protease inhibitor cocktail) were added to this powder. The mixture underwent sonication three times on ice, employing a high-intensity ultrasonic processor (Scientz). Subsequently, the debris was eliminated through centrifugation at 12,000 g at 4 °C for 10 minutes. The resulting supernatant was collected, and the protein concentration was assessed using a BCA kit following the manufacturer's instructions.

Digestion

To initiate digestion, each sample containing 300 μg of protein was slowly added to reach a final concentration of 20% (m/v) TCA for protein precipitation. The mixture was vortexed for thorough mixing and then incubated at 4 °C for 2 hours. The resulting precipitate was collected through centrifugation at 4,500 g for 5 minutes at 4 °C. The precipitated protein underwent three washes with 1 mL of pre-cooled acetone. Subsequently, the precipitate was dispersed using 300 μL of 200 mM TEAB through ultrasonication. After that, trypsin was added at a 1:50 trypsin-to-protein mass ratio and left overnight. The sample was reduced with 5 mM dithiothreitol for 60 minutes at 37 °C and then alkylated with 11 mM iodoacetamide for 45 minutes at room temperature in darkness. Finally, the peptides were desalted using a Strata X SPE column.

TMT labeling

Initially, tryptic peptides were dissolved in 0.5 M TEAB solution. Subsequently, each channel of peptides was labeled with the respective TMT reagent according to ThermoFisher Scientific's protocol and incubated at room temperature for 2 hours. Five microliters from each sample were pooled and subjected to desalting, followed by analysis using mass spectrometry to assess labeling efficiency. Once labeling efficiency was confirmed, samples were quenched by adding 5% hydroxylamine. The pooled samples were then desalted using a Strata X C18 SPE column (Phenomenex) and dried via vacuum centrifugation.

HPLC fractionation

The sample was fractionated using high pH reverse-phase HPLC with an Agilent 300Extend C18 column (5 μm particles, 4.6 mm ID, 250 mm length). The wavelength was set to 214 nm, and the column oven temperature was maintained at 35°C with 95% buffer A (2% ACN, pH 9.0 adjusted by ammonia) and 5% buffer B (98% ACN, pH 9.0 adjusted by ammonia) for at least 30 minutes. Once a flat baseline was obtained, 1 mL of buffer A was added to the peptide sample, which was then vortexed for dissolution. After centrifugation at 12,000 g for 5 minutes, the supernatant was transferred to a new tube. The sample was centrifuged again, the supernatant collected, and loaded onto the HPLC. The separation process was initiated, and simultaneously, the automatic collector was activated. The samples were collected at 1 min per tube from the 9th to the 72nd tube, resulting in a total of 64 tubes. Finally, the peptides from these tubes were combined into 6 fractions and dried using vacuum centrifugation.

Supplementary Note 2: Mass Spectrometry Analysis

Mass spectrometer

The tryptic peptides were dissolved in solvent A and then directly loaded onto a custom-made reversed-phase analytical column (25 cm length, 100 µm i.d.). The mobile phase, comprised of solvent A (0.1% formic acid, 2% acetonitrile in water) and solvent B (0.1% formic acid, 90% acetonitrile in water), facilitated peptide separation using the following gradient: 0-4 min, 7%-11% B; 4-53 min, 11%-32% B; 53-57 min, 32%-80% B; 57-60 min, 80% B. This separation process occurred at a constant flow rate of 500 nL/min on an EASY-nLC 1,200 UPLC system (ThermoFisher Scientific).

Analysis

After separation, the peptides were analyzed using an Orbitrap Exploris 480 mass spectrometer (ThermoFisher Scientific) equipped with a nano-electrospray ion source. An electrospray voltage of 2,300 V was applied, and FAIMS compensation voltage (CV) was set at -45 V. Both precursors and fragments were examined at the Orbitrap detector. Full MS scans were conducted at a resolution of 60,000, covering the mass-to-charge ratio (m/z) range of 400 and 1,200. The MS/MS scans were initiated using TurboTMT with a fixed first mass of 110 m/z and a resolution of 15,000. Subsequently, up to 25 most abundant precursors were chosen for further MS/MS analyses, with a dynamic exclusion duration of 30 seconds. HCD fragmentation was executed at a normalized collision energy (NCE) of 35%. The automatic gain control (AGC) target was set to 100%, with an intensity threshold of 10000 ions/s and an Auto maximum injection time.

Database searching

The obtained MS/MS data were analyzed using Proteome Discoverer software (version 2.4.1.15). Tandem mass spectra were searched in the *Rattus norvegicus* database (containing 29,934 entries) combined with a reverse decoy database. Trypsin/P was chosen as the cleavage enzyme, allowing up to 2 missing cleavages. The mass tolerance for precursor ions was set at 20 ppm in the initial search and 5 ppm in the main search, while the mass tolerance for fragment ions was set at 0.02 Da. Carbamidomethyl on cysteine was considered a fixed modification, and acetylation on protein N-terminal and oxidation on methionine were treated as variable modifications. To ensure data falsifiability, the false discovery rate (FDR) was controlled at less than 1%.

Supplementary Note 3: Bioinformatics tools and databases

Gene Ontology (GO) database

The Gene Ontology (GO) database is a major bioinformatics initiative that aims to develop a computational representation of our evolving knowledge of how genes encode biological functions at the molecular, cellular, and tissue system levels. It is the world's largest source of information on the functions of genes. This knowledge is both human-readable and machine-readable, and serves as a foundation for computational analysis of large-scale molecular biology and genetics experiments in biomedical research. Gene Ontology describes knowledge of the biological domain with respect to three aspects: molecular function (MF), biological process (BP), and cellular component.

Molecular function refers to the molecular-level activities performed by gene products. Molecular function terms describe activities that occur at the molecular level, such as *catalysis* or *transport*. These functions generally correspond to activities that can be performed by individual gene products, but some activities are performed by molecular complexes composed of multiple gene products. Examples of broad functional terms are catalytic activity and transporter activity; examples of narrower functional terms are *adenylate cyclase activity* or *Toll-like receptor binding*. To avoid confusion between gene product names and their molecular functions, GO molecular functions are often appended with the word *activity* (a protein kinase would have the GO molecular function protein kinase activity).

Biological process represents the larger processes, or "biological programs" accomplished by multiple molecular activities. Examples of broad biological process terms are *DNA repair* or *signal transduction*. More specific terms might include *pyrimidine nucleobase biosynthetic process* or *glucose transmembrane transport*. At present, the GO does not try to represent the dynamics or dependencies that would be required to fully describe a pathway.

Cellular component refers to the locations relative to cellular structures in which a gene product performs a function. These can be either cellular compartments (e.g., *mitochondrion*) or stable macromolecular complexes of which they are parts (e.g., the *ribosome*). Unlike the other aspects of GO, cellular component classes refer not to processes but rather a cellular anatomy.

GO terms are assigned to gene products through *annotation*. GO annotations for gene products can change over time as the evolvement of the understanding of gene functions. Annotation involves associating each gene product with the appropriate GO terms that describe its molecular function, biological process, and cellular component. The annotation process is carried out by members of the GO Consortium (GOC), which is a community-based bioinformatics project. The GOC uses structured controlled vocabularies to classify gene product function.

KEGG PATHWAY

The Kyoto Encyclopedia of Genes and Genomes (KEGG) is a database resource that aims to understand high-level functions and utilities of the biological system, such as the cell, the organism, and the ecosystem, from molecular-level information. One of the most important and commonly used sub-databases in KEGG is the KEGG PATHWAY. It is a collection of manually drawn pathway

maps representing our knowledge of the molecular interaction, reaction, and relation networks. These pathway maps are created by researchers based on existing research literature.

KEGG PATHWAY database covers seven major categories, which are, metabolism, genetic information processing, environmental information processing, cellular process, organismal systems, human diseases, and drug development. These pathway maps are created based on existing research literature, making them a reliable source of information.

KEGG PATHWAY database is widely used by bioinformaticians. It provides a comprehensive knowledge base for understanding high-level functions and utilities of the biological system, from molecular-level information to organismal systems. This makes it a valuable resource for interpreting genome sequences and other high-throughput data.

Gene set enrichment analysis (GSEA)

Gene Set Enrichment Analysis (GSEA) is a computational approach designed to determine whether a predefined set of genes exhibits statistically significant, consistent differences between two biological states (e.g. phenotypes). It is a major bioinformatics initiative to develop a computational representation of our evolving knowledge of how genes encode biological functions at the molecular, cellular and tissue system levels.

GSEA utilizes statistical techniques to identify gene groups that are significantly enriched or depleted. Researchers performing high-throughput experiments that yield sets of genes often want to retrieve a functional profile of that gene set, in order to better understand the underlying biological processes.

GSEA method employs a weighted procedure where increments to the running sum are proportionate to the rank metric for the gene set. The process begins by ranking all genes in the dataset based on their correlation with a specified phenotype. Various metrics measuring the association between gene expression and the phenotype can be utilized for this purpose, resulting in a ranked gene list. Subsequently, the enrichment score (ES) is computed by traversing the ranked gene list. If a gene belongs to the gene set of interest, the running-sum statistic increases; conversely, if it does not, the statistic decreases. The magnitude of this increment is contingent upon the correlation of the gene with the phenotype. The ES represents the maximum deviation from zero encountered during this traversal and corresponds to a weighted Kolmogorov-Smirnov-like statistic.

A permutation test is performed to estimate the significance level of the ES. This entails shuffling the phenotype labels, preserving gene-gene correlations. The ES is recalculated for each permutation, creating a null distribution. The nominal *p*-value of the observed ES is then calculated relative to this null distribution, providing an estimation of its significance. Given the multitude of gene sets tested for enrichment in GSEA, it becomes imperative to correct for multiple hypothesis testing to prevent false positives. This correction is achieved by calculating a False Discovery Rate (FDR) using Benjamini-Hochberg correction corresponding to each ES.

Protein-protein interaction and STRING database

Proteins rarely act alone as their functions tend to be regulated. Many molecular processes within a cell are carried out by molecular machines that are built from numerous protein components

organized by their protein-protein interactions (PPIs). These physiological interactions make up the so-called interactomes of the organism, while aberrant PPIs are the basis of multiple aggregation-related diseases, such as Creutzfeldt-Jakob and Alzheimer's diseases.

PPIs are physical contacts of high specificity established between two or more protein molecules as a result of biochemical events steered by interactions that include electrostatic forces, hydrogen bonding, and the hydrophobic effect.

PPIs have been studied with many methods and from different perspectives, such as biochemistry, quantum chemistry, molecular dynamics, signal transduction, among others. All this information enables the creation of large protein interaction networks that empower the current knowledge on biochemical cascades and molecular etiology of disease, as well as the discovery of putative protein targets of therapeutic interest.

PPI network is an important ingredient for the system-level understanding of cellular processes. Such networks can be used for filtering and assessing functional genomics data and for providing an intuitive platform for annotating structural, functional, and evolutionary properties of proteins. Exploring predicted interaction networks can suggest new directions for future experimental research and provide cross-species predictions for efficient interaction mapping. Therefore, one of the widely used databases is STRING.

The interactions in STRING include both direct (physical) and indirect (functional) associations. They stem from computational prediction, from knowledge transfer between organisms, and from interactions aggregated from other (primary) databases. It contains information from numerous sources, including experimental data, computational prediction methods, and public text collections. It is freely accessible, and it is regularly updated. The resource also serves to highlight functional enrichments in user-provided lists of proteins, using several functional classification systems such as GO, Pfam, and KEGG. Therefore, various computational predictions can be inspected from different designated views.

t-distributed Stochastic Neighbor Embedding (t-SNE)

t-distributed Stochastic Neighbor Embedding (t-SNE) is a statistical method for visualizing high-dimensional data by giving each datapoint a location in a two or three-dimensional map. It is often used to visualize complex datasets into two and three dimensions, allowing us to understand more about underlying patterns and relationships in the data.

The t-SNE algorithm involves two main stages. First, t-SNE constructs a probability distribution over pairs of high-dimensional objects in such a way that similar objects are assigned a higher probability while dissimilar points are assigned a lower probability. Then, t-SNE defines a similar probability distribution over the points in the low-dimensional map, and it minimizes the Kullback-Leibler divergence (KL divergence) between the two distributions with respect to the locations of the points in the map.

Both t-SNE and principal component analysis (PCA) are dimensional reduction techniques that have different mechanisms and work best with different types of data. PCA is a linear technique that works best with data that has a linear structure. It seeks to identify the underlying principal components in the data by projecting onto lower dimensions, minimizing variance, and preserving

large pairwise distances. But t-SNE is a nonlinear technique that focuses on preserving the pairwise similarities between data points in a lower-dimensional space. t-SNE is concerned with preserving small pairwise distances, whereas PCA focuses on maintaining large pairwise distances to maximize variance.

Supplementary Figure 1

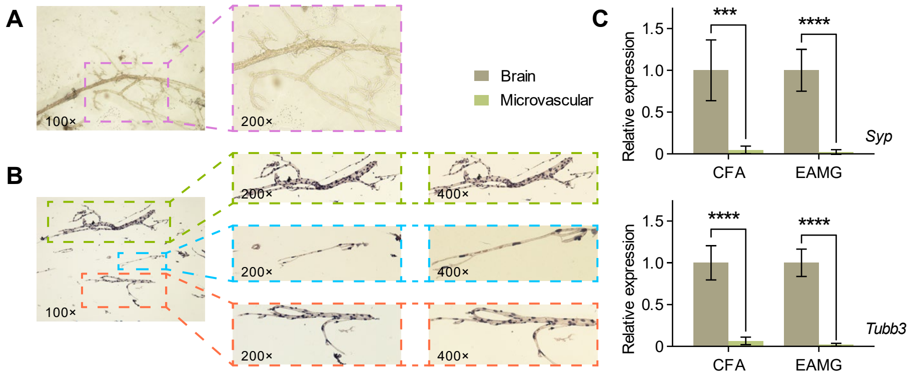

Cerebral microvascular extraction and identification in CFA and EAMG rats. The cerebral microvascular was confirmed by the tubular structure via (A) optical microscope and (B) HE staining. (C) the absence of the neuronal markers (*Syp* and *Tubb3* genes) via qPCR. Statistical analysis was performed using *t* test, *** $P < 0.001$, **** $P < 0.0001$.

Supplementary Figure 2

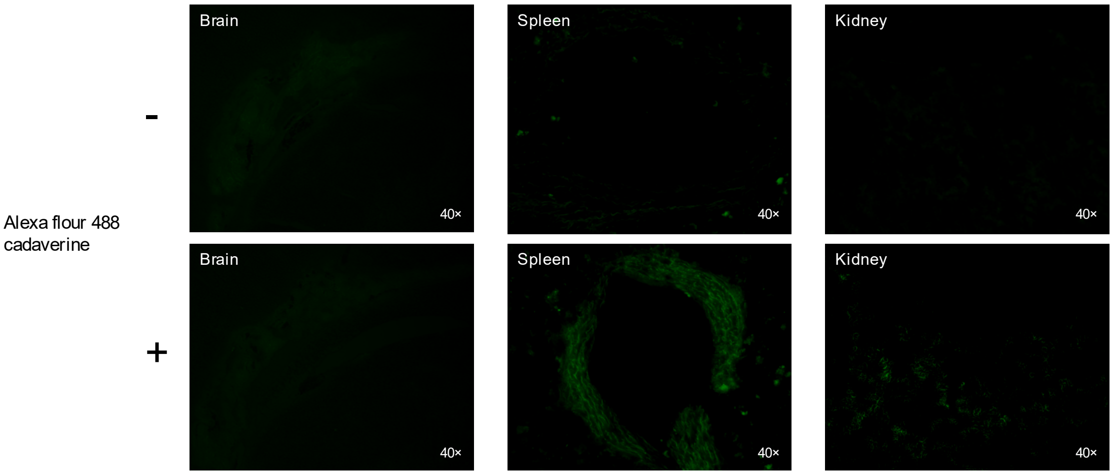

The leakage of BBB in EAMG rats. The brain, spleen and kidney fluorescence, "+" and "-" indicated with or without Alexa flour 488 cadaverine injection (40 × magnification).

Supplementary Figure 3

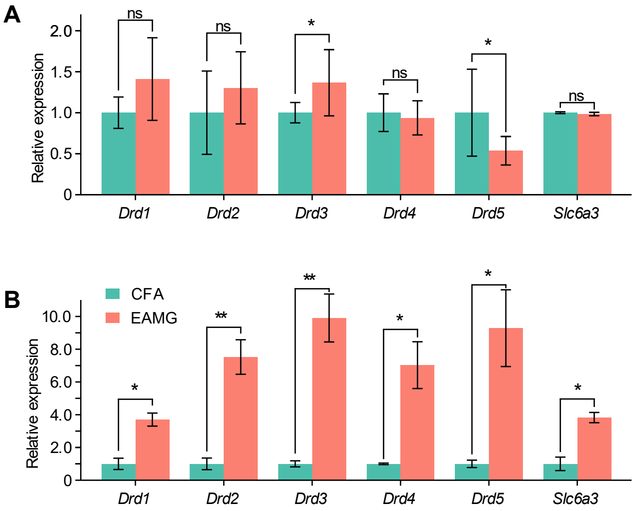

The expression of *DRD1-DRD5* and *Scl6a3* genes in T cells (A) and B cells (B) of CFA and EAMG groups. Statistical analysis was conducted using *t* test, * $P < 0.05$, ** $P < 0.01$, ns = no significance.

Supplementary Figure 4

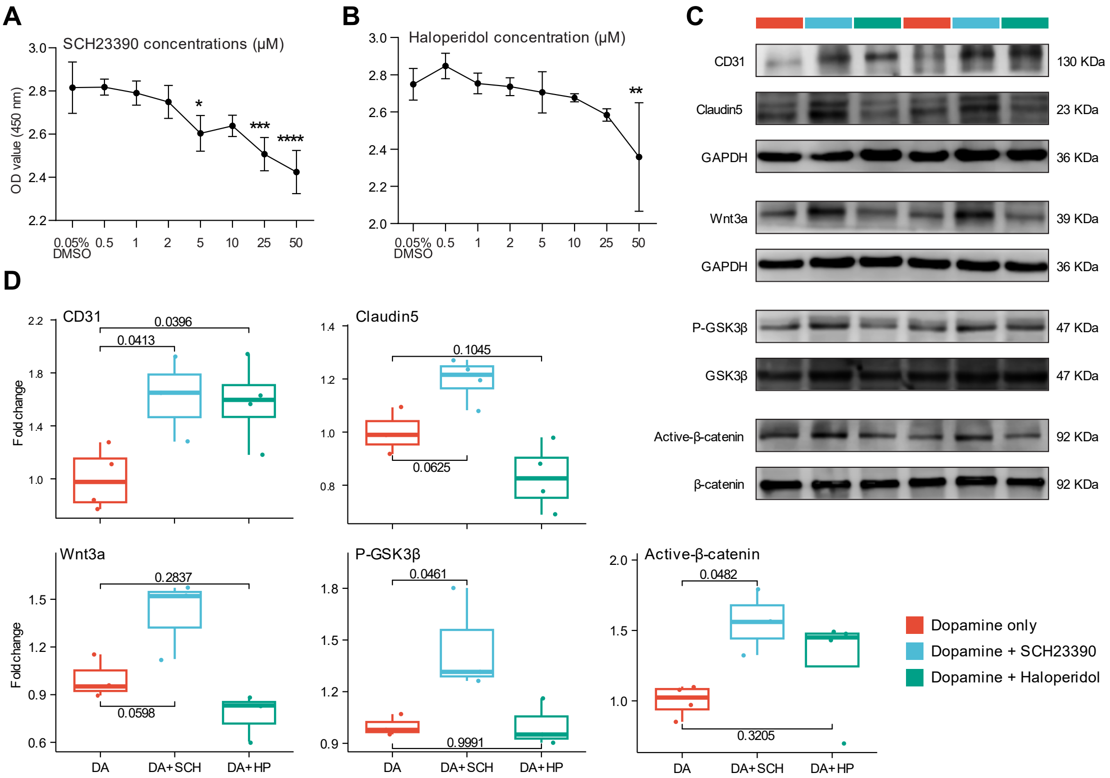

The impact of SCH23390 (dopamine D1 receptor antagonist) and Haloperidol (dopamine D2 receptor antagonist) on bEnd.3 cells was investigated. (A-B) The proliferative effects of SCH23390 and Haloperidol on bEnd.3 cell line were assessed using CCK-8 assays. (C-D) The expression of CD31 was significantly increased upon incubation with both SCH23390 and Haloperidol, while Claudin5 remained unchanged. Additionally, the expression levels of Wnt3a in bEnd.3 cell line were examined through western blot, showing no significant changes in SCH23390 and Haloperidol-treated cell lines. However, the results examined through western blot also showed elevated levels of p-GSK3β and active-β-catenin in SCH23390-treated cell line. Statistical analysis was conducted using one-way ANOVA, * $P < 0.05$, ** $P < 0.01$, *** $P < 0.001$, **** $P < 0.0001$, ns = no significant difference.

Supplementary Figure 5

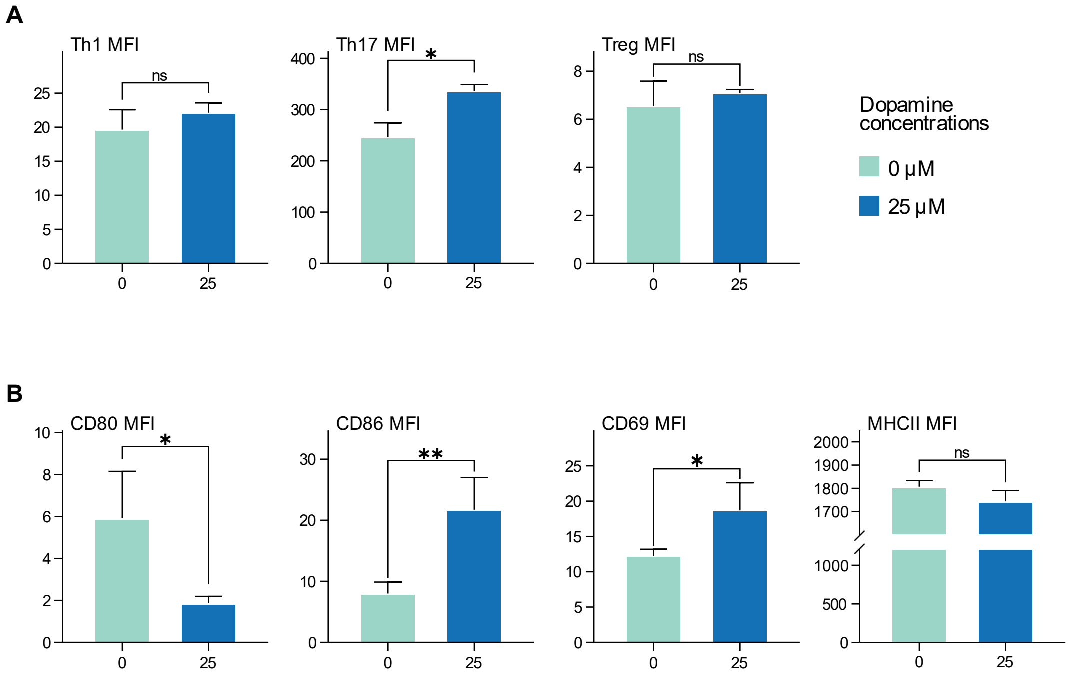

The effects of dopamine on T and B cells were examined. (A) Upon dopamine incubation, the mean fluorescence intensity (MFI) level of Th17 cells were significantly increased, whereas the MFI of Th1 and Treg cells exhibited no significant changes. (B) The dopamine exposure resulted in a significant increase in the MFI of $CD86^+$ B cells and $CD69^+$ B cells. Conversely, the MFI of $CD80^+$ B cells decreased, and $MHCII^+$ B cells remained unsignificant. Statistical analysis was conducted using *t* test, * $P < 0.05$, ** $P < 0.01$, ns = no significant difference.

Supplementary Figure 6

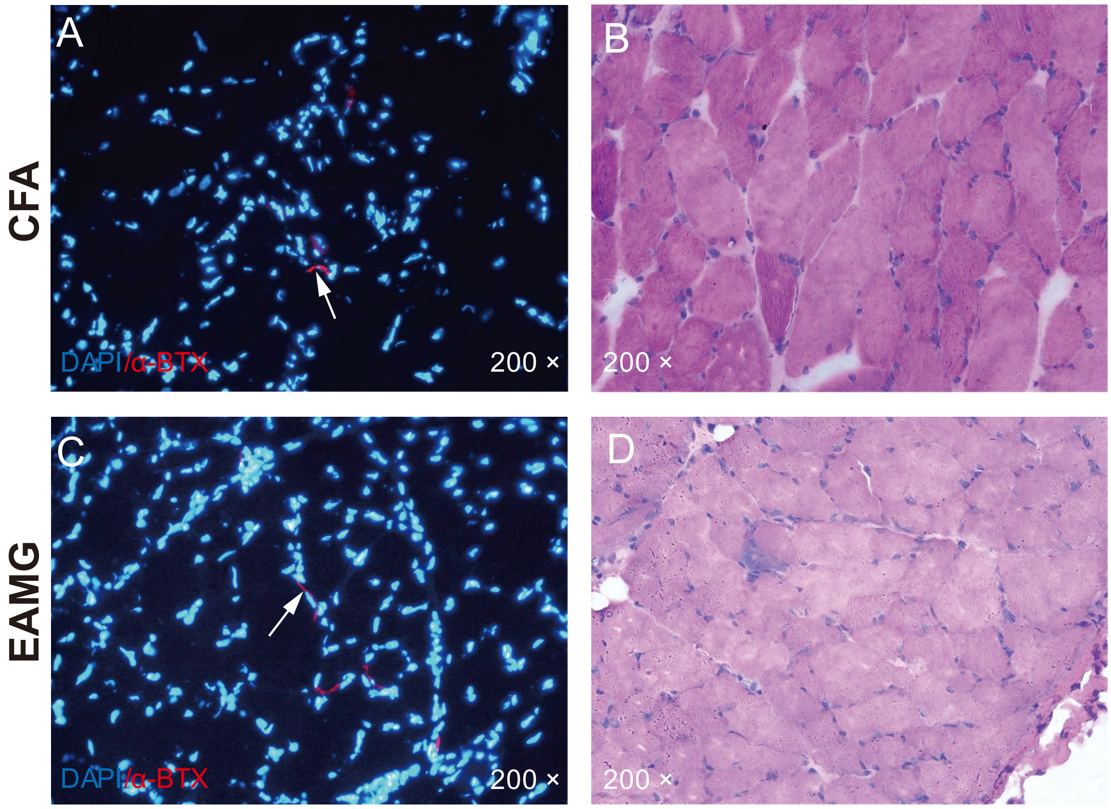

Diaphragm samples were analyzed using immunofluorescence and HE staining. We performed (A) α-BTX and DAPI staining and (B) HE staining in the CFA group. We also performed (C) α-BTX and DAPI staining and (D) HE staining in the EAMG group. Blue staining represents DAPI, and α-BTX is the red staining highlighted by the white arrow.

Supplementary Figure 7

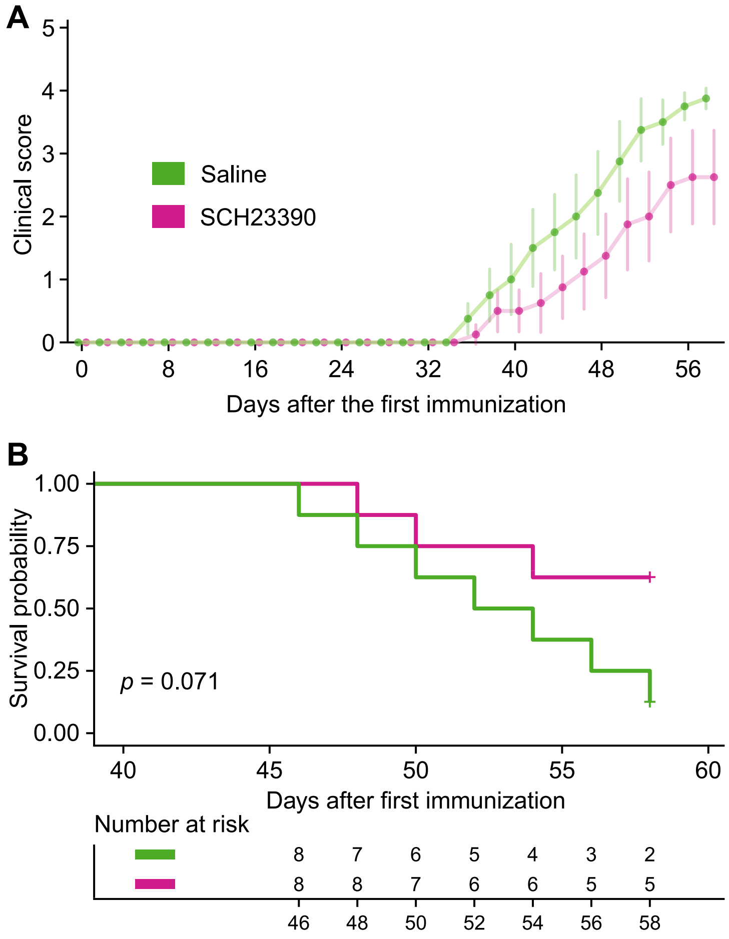

The effect of SCH23390 (dopamine D1 receptor antagonist) on EAMG rats was investigated by gavage every other day after the first immunization *in vivo*. (A) clinical scores. (B) survival probability and number at risk.

Supplementary Table 1

The criterion for each EAMG score is listed below.

Score	Symptoms
0	No disease and normal muscle strength
1	Mildly decreased activity and body weight, weak grip
2	Weakness, fatigue, clinical signs before exercise (tremors, head down, hunched posture)
3	Severe generalized weakness, no grip, moribund
4	Dead

Clinical scores with intermediate signs were scored in average, such as 0.5, 1.5, 2.5 or 3.5.

Supplementary Table 2

The rat primer sequences are listed below.

Name	Forward	Reverse
18s	5’-AGTCCCTGCCCTTTGTACACA-3’	5’-CGATCCGAGGGCCTCACTA-3’
Chgb	5’-GGAGAAGCCAGTCCAACAAG-3’	5’-CTCTGAAGCCCGGTAGTGAG-3’
Th	5’-GGTATTTGAGGAGAGGGATGG-3’	5’-CGAGACAAGGAGGAGGGTTT-3’
Drd1	5’- CCAGCCCTTTCCAGTATGAG-3’	5’-GTTGTCATCCTCGGTGTCCT-3’
Drd2	5’- CTCAGGAGCTGGAAATGGAG-3’	5’- GTTGCTATGTAGGCCGTGGT-3’
Drd3	5’- TAAGCACCCACCTCAACTCC-3’	5’- AGCACAGCAGCACATACCAG-3’
Drd4	5’-TCTCCAGCAGACCAGAGTCA-3’	5’- AGAAAGGCGTCCAACACATC-3’
Drd5	5’- ACACGGTCTTCCACAAGGAG-3’	5’- CACAGTCAAGCTCCCAGACA-3’
Slc6a3	5’- TGGCATCAGAGCATACCTCA-3’	5’- GGAGAAGGCAATCAGCACTC-3’
Foxa2	5’- CAGTGGCGGAGGCAAGAAG -3’	5’- GGTGGTTGAAGGCGTAATGGT -3’
Nurr1	5’- CGATCTCCTGACTGGCTCTAT -3’	5’- CACTGGGTTGGACCTGTATG -3’
Kcnj6	5’- CCTCCATCAGAGCCAAGTT-3’	5’- CACGAGAAAGAGTCGGTCA-3’
Syp	5’- CGCTTTCATGTGGCTAGTTAGTT -3’	5’- TGATTGGAGAAGGAGGTGGG-3’
Tubb3	5’- CCTTCATCGGCAACAGCA -3’	5’- CCTCGTCGTCATCTTCATACATC -3’
Tjp1	5’- AGCGGGCTACCTTATTGA -3’	5’- TGGTTCTGGAAGAGTGGG -3’
Tjp2	5’- AGAAGAACATTCGCAAGAGC-3’	5’- TTTGAAACAGGTCGGGTAG -3’
Pecam1	5’- AAGTGGAGTCCAGCCGCATATC-3’	5’- ATGGAGCAGGACAGGTTCAGTC-3’
Gja1	5’- GCTGGTGGTGTCCTTGGT -3’	5’- AGTGGAGCCGTTGGTGAG-3’
Cldn5	5’- TGTCTGGTAGGCTGGGTGGG -3’	5’- ACATGAGCAGTGCGGAGGC -3’
Ocln	5’- TGCTTCATCGCTTCCTTG -3’	5’- CCATCTTTCTTCGGGTTTT -3’
Aqp4	5’- GCCAAGTCCGTCTTCTACATC -3’	5’- ACTCCCAATCCTCCCACC-3’
Gfap	5’- ATGACTATCGCCGCCAACT-3’	5’- CTCGCCATCCCGCATCT-3’
Pdgfrb	5’- ACCACGGCGATGAGAAAG -3’	5’- ACAGAGGGCGTCGGATAA -3’
Anpep	5’- CGATGGATTCTACAGATGGGTT -3’	5’- CGCACGGTTGATGACAGG -3’
Cspg4	5’- CTTCTCCGCCCACTAACCC -3’	5’- AGTTGCCACGCTTGTCCC -3’

Supplementary Table 3

The mouse primer sequences are listed below.

Name	Forward	Reverse
Tjp1	5'- TTCCTCCTTGACCTCCC -3'	5'- ATCACCACCCGCTGTCT -3'
Tjp2	5'- CAAGTTCCCTGCCTACGAGA -3'	5'- CTGCTGCTGAATGCTGTCC -3'
Pecam1	5'- CTGCCAGTCCGAAAATGGAAC -3'	5'- CTTCATCCACCGGGGCTATC -3'
Gja1	5'- ACAGCGGTTGAGTCAGCTTG -3'	5'- GAGAGATGGGGAAGGACTTGT -3'
Cldn5	5'- ATTGAGACAGACCCCAAACG -3'	5'- TTCTGGTTTTCTGGCAGCTT -3'
Ocln	5'- TTGAAAGTCCACCTCCTTACAGA -3'	5'- CCGGATAAAAAGAGTACGCTGG -3'

Supplementary Table 4

The detailed statistical results.

Figure	x axis	Mean diff	*t*	*df*	*p*	R^2
1A	Day 36	-0.2000	-1.413	450	0.9866	
	Day 38	-0.4000	-2.825	450	0.1163	
	Day 40	-1.000	-7.063	450	<0.0001	
	Day 42	-1.300	-9.182	450	<0.0001	
	Day 44	-1.900	-13.42	450	<0.0001	
	Day 46	-2.800	-19.78	450	<0.0001	
	Day 48	-3.200	-22.60	450	<0.0001	
1B	Day 0	2.600	0.6819	442	1	
	Day 2	1.580	0.4144	442	1	
	Day 4	0.2700	0.07081	442	1	
	Day 6	-0.3500	-0.09179	442	1	
	Day 8	1.620	0.4249	442	1	
	Day 10	1.810	0.4747	442	1	
	Day 12	2.420	0.6347	442	1	
	Day 14	3.280	0.8602	442	1	
	Day 16	4.170	1.094	442	0.9997	
	Day 18	3.140	0.8235	442	1	
	Day 20	2.230	0.5848	442	1	
	Day 22	3.240	0.8497	442	1	
	Day 24	3.350	0.8786	442	1	
	Day 26	2.560	0.6714	442	1	
	Day 28	1.520	0.3986	442	1	
	Day 30	3.210	0.8419	442	1	
	Day 32	2.160	0.5665	442	1	
	Day 34	3.120	0.8183	442	1	
	Day 36	4.650	1.220	442	0.9982	
	Day 38	6.550	1.718	442	0.8959	
	Day 40	11.28	2.958	442	0.0784	
	Day 42	20.27	5.316	442	<0.0001	
	Day 44	28.66	7.316	442	<0.0001	
	Day 46	42.82	10.19	442	<0.0001	
	Day 48	54.86	12.46	442	<0.0001	
1D		-0.5240	-18.10	5	<0.0001	0.8909
1E		0.7069	8.081	8	<0.0001	0.9850
2B	TH	0.5077	2.804	6	0.0310	0.5672
	CHGB	0.7177	6.245	5	0.0015	0.8864

Figure	x axis	Mean diff	*t*	*df*	*p*	R^2
2C	*Th*	0.6043	3.657	16	0.0021	0.4552
	Chgb	0.3791	4.309	6	0.0050	0.7558
2E	*Foxa2*	1.610	3.550	10	0.0053	0.5576
	Kcnj6	0.5938	4.618	12	0.0006	0.6399
	Nurr1	0.01120	0.1055	7	0.9189	0.001589
2F	*Drd1*	0.7124	2.782	9	0.0213	0.4624
	Drd2	0.09471	0.6873	15	0.5024	0.03053
	Drd3	0.3639	2.204	19	0.0401	0.2036
	Drd4	0.1415	0.9516	12	0.3601	0.07017
	Drd5	0.6099	2.236	12	0.0451	0.2941

	Slc6a3	0.7851	3.800	12	0.0025	0.5461
4B	*Aqp4*	-0.4501	-2.997	12	0.0111	0.4280
	Gfap	-0.2975	-4.283	19	0.0004	0.4912
4C	*Pdgfrb*	-0.1308	-0.4794	13	0.6396	0.01737
	Cspg4	-0.1810	-1.280	8	0.2365	0.1699
	Anpep	-0.3474	-2.615	6	0.0399	0.5326
4D	*Tjp1*	-0.01810	-0.08506	5	0.9355	0.001445
	Tjp2	-0.5948	-3.674	7	0.0079	0.6586
	Ocln	0.09939	0.6207	10	0.5487	0.03710
	Cldn5	-0.4307	-3.136	9	0.0120	0.5221
	Gja1	-0.3262	-3.570	7	0.0091	0.6455
	Pecam1	-0.6200	-3.988	8	0.0040	0.6653
4F	ZO2	-0.3248	-1.996	6	0.0930	0.3990
	Occludin	-0.1371	-1.999	4	0.1162	0.4998
	Claudin5	-0.7290	-8.215	6	0.0002	0.9184
	CD31	-0.5197	-3.098	5	0.0269	0.6574
5A		0.08533	6.912	4	0.0023	0.9228
5B	0.5	-0.1582	-0.1582	16	0.1146	
	2.5	-0.1170	-0.1170	16	0.3359	
	5	-0.4473	-0.4473	16	<0.0001	
	10	-0.3048	-0.3048	16	0.0012	
	25	-0.01593	-0.01593	16	>0.9999	
	50	0.2233	0.2233	16	0.0159	
	100	1.142	1.142	16	<0.0001	
5D	ZO1	-0.1765	-3.224	4	0.0322	0.7221
	CD31	-0.3869	-4.420	6	0.0045	0.7651
	Occludin	0.03326	0.3477	4	0.7456	0.02933
	CXC43	-0.1118	-0.5039	6	0.6323	0.04060
	Claudin5	-0.3641	-2.450	6	0.0498	0.5001
5E	*Tjp1*	-0.4421	-3.434	9	0.0075	0.5672

Figure	x axis	Mean diff	*t*	*df*	*p*	R^2
5E	*Tjp2*	-0.3373	-4.494	8	0.0020	0.7162
	Ocln	0.05543	-0.5061	11	0.6227	0.02276
	Cldn5	-0.4356	-2.637	7	0.0336	0.4983
	Pecam1	-0.2202	-2.492	10	0.0319	0.3832
	Gja1	-0.03659	-0.4378	11	0.6700	0.01712
6B	Wnt3a	-0.4315	-9.008	2	0.0121	0.9759
6C	GSK3β	0.04058	-0.3873	4	0.7183	0.03614
	P-GSK3β	-0.3206	-3.385	5	0.0196	0.6962
6D	β-catenin	0.007036	0.09888	6	0.9245	0.001627
	Active-β-catenin	-0.3839	-4.429	4	0.0114	0.8306
7A	0.5 μM	0.5300	4.001	16	0.0058	
	2.5 μM	0.4870	3.677	16	0.0112	
	5 μM	-0.05293	-0.3996	16	0.9986	
	10 μM	-0.4214	-3.181	16	0.0302	
	25 μM	-0.2594	-1.958	16	0.2786	
	50 μM	0.8992	6.788	16	<0.0001	
	100 μM	1.688	12.74	16	<0.0001	
7B	0.5 μM	0.001367	0.08511	16	>0.9999	
	2.5 μM	-0.1160	-7.224	16	<0.0001	
	5 μM	-0.1260	-7.845	16	<0.0001	
	10 μM	-0.1486	-9.256	16	<0.0001	
	25 μM	-0.1369	-8.524	16	<0.0001	

	50 μM	-0.1461	-9.101	16	<0.0001	
	100 μM	-0.04057	-2.526	16	0.1057	
7C	IFN-γ	-0.6225	-3.342	6	0.0156	0.6505
	IL-17A	-4.020	-5.162	4	0.0067	0.8695
	Foxp3	-0.7167	-1.115	4	0.3274	0.2371
7D	CD138	-1.047	-6.294	5	0.0015	0.8879
	CD80	0.1675	0.6844	6	0.5193	0.07241
	CD86	-0.6650	-1.812	5	0.1298	0.3962
	MHCII	-1.150	-5.535	5	0.0026	0.8597
	CD69	-1.209	-3.069	5	0.0278	0.6533
8A	ZO1	0.02677	0.1965	23	>0.9999	0.001676
	ZO2	0.2829	1.857	23	0.3784	0.1304
	CXC43	0.5362	3.935	23	0.0040	0.4024
	Claudin5	0.3064	2.248	23	0.1896	0.1801
	Occludin	0.1619	1.188	23	0.8177	0.05782
	CD31	0.5057	3.711	23	0.0069	0.3745
8B	*Tjp1*	0.1731	0.9117	3	0.4291	0.2170
	Tjp2	0.3543	3.079	5	0.0275	0.6547

Figure	x axis	Mean diff	*t*	*df*	*p*	R^2
8B	*Gja1*	0.3476	2.295	3	0.1055	0.6371
	Cldn5	0.2021	2.328	3	0.1024	0.6437
	Ocln	0.5065	1.844	4	0.1389	0.4595
	Pecam1	0.1114	0.4592	3	0.6773	0.06567
8C	ZO1	-0.2841	2.312	18	0.1538	0.2290
	ZO2	0.7058	5.743	18	<0.0001	0.6469
	CXC43	-0.2701	2.458	18	0.1160	0.2513
	Claudin5	0.06059	0.5513	18	0.9882	0.01660
	Occludin	0.2905	2.643	18	0.0800	0.2796
8D	*Tjp1*	0.09118	0.4120	4	0.7015	0.04071
	Tjp2	0.4003	3.299	4	0.0300	0.7312
	Gja1	0.3730	1.611	4	0.1824	0.3935
	Cldn5	0.2287	1.192	4	0.2990	0.2621
	Ocln	0.2670	0.8931	5	0.4127	0.1376
	Pecam1	-0.2015	-0.6068	6	0.5662	0.05782
8E	ZO2	-0.3070	-0.8808	4	0.4282	0.1624
	Claudin5	0.02097	0.07494	4	0.9439	0.001402
	Occludin	0.03323	0.3249	4	0.7616	0.02571
	CD31	0.7552	3.556	3	0.0379	0.8083
8F	*Tjp1*	0.05469	0.3768	34	0.9979	0.004158
	Tjp2	0.8074	5.303	34	<0.0001	0.4527
	Cldn5	0.3164	1.842	34	0.3199	0.09074
	Ocln	0.3540	2.156	34	0.1773	0.1203
	Pecam1	0.6982	4.391	34	0.0005	0.3619
S1C	*Syp*-CFA	0.9515	7.407	14	<0.0001	0.7967
	Syp-EAMG	0.9725	10.92	13	<0.0001	0.9017
	Tubb3-CFA	0.9347	12.53	14	<0.0001	0.9181
	Tubb3-EAMG	0.9808	17.17	12	<0.0001	0.9609
S3A	*Drd1*	-0.4078	-1.878	13	0.0830	0.2134
	Drd2	-0.3022	-1.315	15	0.2083	0.1034
	Drd3	-0.3671	-2.449	14	0.0281	0.3000
	Drd4	0.06153	0.5741	15	0.5744	0.02150
	Drd5	0.4653	2.237	14	0.0421	0.2633
	Slc6a3	0.01526	1.913	17	0.0728	0.1771

S3B	*Drd1*	-2.128	-3.234	6	0.0178	0.6355
	Drd2	-7.337	-4.964	6	0.0025	0.8042
	Drd3	-8.428	-5.358	6	0.0017	0.8271
	Drd4	-4.422	-3.045	4	0.0382	0.6986
	Drd5	-7.793	-3.094	6	0.0213	0.6147
	Slc6a3	-2.728	-3.531	6	0.0123	0.6752

Figure	x axis	Mean diff	t	df	p	R^2
S4A	0.5	0.002133	0.03292	20	>0.9999	
	1	-0.02520	0.3889	20	0.9998	
	2	-0.06643	1.025	20	0.8876	
	5	-0.2118	3.269	20	0.0253	
	10	-0.1770	2.732	20	0.0772	
	25	-0.3084	4.759	20	0.0009	
	50	-0.3910	6.034	20	<0.0001	
	0.5	0.09793	1.064	20	0.8681	
	1	0.004033	0.04383	20	>0.9999	
	2	-0.01310	0.1424	20	>0.9999	
	5	-0.04360	0.4738	20	0.9990	
	10	-0.07257	0.7886	20	0.9700	
	25	-0.1653	1.797	20	0.3974	
	50	-0.3912	4.251	20	0.0029	
S4D	CD31 – 2	0.6188	2.806	8	0.0413	
	CD31 – 3	0.5786	2.833	8	0.0396	
	Claudin5 – 1	0.1965	2.510	8	0.0625	
	Claudin5 – 2	-0.1698	2.169	8	0.1045	
	Wnt3a – 1	0.4055	2.723	6	0.0598	
	Wnt3a – 2	-0.2292	1.540	6	0.2837	
	P-GSK3β – 1	0.4599	2.926	6	0.0461	
	P-GSK3β – 2	-0.005482	0.03487	6	0.9991	
	Active-β-catenin – 1	0.5608	2.704	8	0.0482	
	Active-β-catenin – 2	0.2716	1.415	8	0.3205	
S5A	Th1 MFI	2.475	1.519	6	0.2743	0.2778
	Th17 MFI	89.83	5.184	3	0.0139	0.8996
	Treg MFI	0.5400	0.8914	4	0.4231	0.1657
S5B	CD80 MFI	-4.047	3.687	5	0.0142	0.7311
	CD86 MFI	13.73	4.930	5	0.0044	0.8294
	CD69 MFI	6.450	3.264	5	0.0223	0.6806
	MHCII MFI	-63.33	2.095	5	0.0903	0.4675

Supplementary Table 5

Materials and reagents required for sample preparation.

Name	Company
**Protein extraction**	
Urea	Sigma
SDS	Sigma
Cocktail III	Merck Millipore
BCA kit	Beyotime
**Digestion**	
Trichloroacetic acid (TCA)	Sigma
Acetone	Hangzhou Hannuo
Triethylammonium bicarbonate buffer (TEAB)	Sigma
Trypsin	Promega
Dithiothreitol (DTT)	Sigma
iodoacetamide (IAM)	Sigma
H_2O	ThermoFisher Scientific
Trifluoroacetic acid (TFA)	Sigma-Aldrich
Methanol	ThermoFisher Scientific
Strata X SPE	Phenomenex
**TMT Labeling**	
TMT reagent	ThermoFisher Scientific
ACN (acetonitrile)	Fisher Chemical
TFA (trifluoroacetic acid)	Sigma-Aldrich
TEAB (Triethylammonium bicarbonate buffer)	Sigma-Aldrich
H_2O	Fisher Chemical
Hydroxylamine	ThermoFisher Scientific
**HPLC fractionation**	
ACN (acetonitrile)	Fisher Chemical
